# Supplementary material for: A spin-refrigerated cavity quantum electrodynamic sensor
Source: Nat Commun. 2024 Nov 28;15:10320. doi: 10.1038/s41467-024-54333-8 (PMC11605127; doi:10.1038/s41467-024-54333-8)
Supplement: Supplementary file 1 — Supplementary Information [file 41467_2024_54333_MOESM1_ESM.pdf]

Supplementary Information of "A spin-refrigerated cavity quantum electrodynamic sensor"

Hanfeng Wang[1], Kunal L. Tiwari[2], Kurt Jacobs[3,4], Michael Judy[5],
Xin Zhang[5], Dirk R. Englund[1,*] and Matthew E. Trusheim[1,3†]
[1] *Massachusetts Institute of Technology,*
50 Vassar Street, Cambridge, MA 02139, USA
[2] *MIT Lincoln Laboratory, Lexington, MA 02421, USA*
[3] *DEVCOM Army Research Laboratory, Adelphi, MD 20783, USA*
[4] *Department of Physics, University of Massachusetts Boston, MA 02125, USA*
[5] *Analog Devices, Inc., 1 Analog Way,*
Wilmington, MA 01887, USA

(Dated: November 1, 2024)

CONTENTS

I. Experimental setup 1

II. Nonlinear Model 2
A. Maxwell-Bloch equations for cavity-NV ensemble system 2
B. Ensemble distribution 4
1. Gaussian distribution 5
2. Lorentzian distribution 6
C. Solution in the Nonlinear Regime 7
1. Approximate treatment of cavity occupancy 7
2. Discussion of full solution 8
3. Bistability Threshold 9
D. Optical polarization cycle and generalized model 11
1. Effective rates for incoherent processes in the $^3A_2$ subspace 11
2. Generalized Maxwell-Bloch equations 13

III. Noise analysis 14
A. Johnson-Nyquist limit 14
B. Phase noise 15
C. Other noise sources 16
D. Model for steady-state cooling in the nonlinear regime 16

IV. Sensor details 17
A. Dynamic range 17
B. Sensor response frequency range 17
C. Test field 18

V. Sensitivity prediction 18

References 20

I. EXPERIMENTAL SETUP

We employ a homodyne circuit to measure the phase change induced by the NV-cavity system as shown in Supplementary Figure 1(a). Probe microwaves from a signal generator are divided into a reference arm and a signal

* englund@mit.edu
† matthew.e.trusheim.civ@army.mil

arm using a Wilkinson microwave power divider. The reference arm is directly connected to the LO port of a mixer (HX3400), with a voltage-controlled phase shifter to tune the relative phase. The signal arm is directed to a circulator with tunable attenuation for power control on the cavity input, and the circulator's microwave output is coupled into the dielectric resonator using a probe loop. The reflected signal from the cavity returns to the circulator and is connected to a low-noise amplifier and subsequently the RF port of the mixer for a homodyne measurement. This setup effectively separates the reflected microwave signal from the incident signal, enabling the measurement of the quadrature part of the reflection coefficient by appropriate setting of the LO phase shifter. The quadrature signal is subsequently digitized using a sampling rate of 200 kS/s.

The diamonds (3 mm × 3 mm × 0.9 mm, 4 ppm NV ensemble, sourced from Element 6) are set at the TE01δ mode maximum point in the center of the dielectric resonator (Skyworks, $\varepsilon_\mathrm{r} \sim 31$). See Supplementary Figure 1(b) and Supplementary Figure 1(c) for cavity mode simulation and reflection measurements. Note that the diamond size is much smaller than the mode homogeneous region so it is fair to assume a homogeneous single spin coupling strength g_s. A 4H-SiC wafer is used for heat transfer and supporting the diamond, while two pieces of low-loss-tangent polytetrafluoroethylene (PTFE) are used to fix and align the dielectric resonator. An aluminum shield is employed to isolate the system from external signals in the lab, such as WiFi and 3G signals (1.9 GHz), and to reduce radiative losses. An 8W 532 nm pump laser is utilized to optically polarize the spin ensemble. External magnetic bias is provided by 3-axis magnetic coils.

In the noise measurements, we first connect the LNA to a 50 Ω resistor to set a baseline for the Johnson-Nyquist limit. Then we restore the cavity and gradually changed the tunable attenuator to test the spin refrigeration with different input microwave power.

We extract parameters from the experimental data with the following procedure

1. We measure the cavity spectrum with spin frequency off-resonance with the cavity frequency to determine the cavity parameters $\kappa_\mathrm{c} = 2\pi \times 130$ kHz, and $\kappa_\mathrm{c1} = 2\pi \times 125$ kHz.

2. We use the two-dimensional reflection coefficient spectrum as a function of Δ and Δ_s in the weak-probe regime to determine the collective coupling $g = 2\pi \times 0.19$ MHz and inhomogeneous linewidth $\Gamma = 2\pi \times 330$ kHz.

3. Using the microwave and laser power dependence, we extract $L = 0.53$, $g_\mathrm{s} = 2\pi \times 18$ mHz, $\gamma_0 = 2\pi \times 26$ kHz assuming an optical excitation cross-section of $\sigma_{^3A_2 \to ^3E} = 7.8 \times 10^{-17}$ cm^2 and spot size of 0.09 cm^2.

II. NONLINEAR MODEL

A. Maxwell-Bloch equations for cavity-NV ensemble system

In this section we provide a description of the nonlinear model used to characterize the experiment. We start with the interaction Hamiltonian between a microwave mode and an NV spin ensemble. We describe the system as an ensemble of two-level systems because the degeneracy of the $m_\mathrm{s} = \pm 1$ NV ground state spin levels is eliminated by applying an external magnetic bias field. This brings one spin transition into proximity with the microwave mode and drive, leaving the other far off resonance. Including the coherent drive, the Hamiltonian can be written as [1, 2]:

$$H = \omega_\mathrm{c} a^\dagger a + \sum \omega_j \sigma_j^\dagger \sigma_j + \sum g_\mathrm{s}(a^\dagger \sigma_j + a\sigma_j^\dagger) + i\sqrt{\kappa_\mathrm{c1}}\beta_\mathrm{in}(a^\dagger e^{-i\omega_\mathrm{d} t} - a e^{i\omega_\mathrm{d} t}), \tag{1}$$

where a is the lowering operator for the cavity field, σ_j is the lowering operator of j-th NV spin, g_s is the single photon coupling strength, ω_c is the cavity frequency, ω_j is the frequency of j-th NV spin, κ_c1 is the cavity input loss, ω_d is the driving frequency. The input microwave drive field, β_in, is related to the power incident on the impedance-matching stub tuner, P, by $|\beta_\mathrm{in}| = L\sqrt{P/\hbar\omega_\mathrm{d}}$. The phenomenological factor, L, accounts for reflection due to impedance mismatch when the NV ensemble is on resonance with the cavity or uncertainty in assumed values for, e.g. optical excitation cross section, and has been estimated in analogous experiments to be $L \approx 0.14$ [3]. Comparison with our measurements yields $L \approx 0.53$.

The inhomogeneous NV ensemble is assumed to have transverse relaxation rate $\gamma/2$ and optically-induced polarization rate γ_p. The cavity system is assumed to be characterized by total loss rate κ and input-coupling loss rate κ_c1. Under the semiclassical approximation, assuming that expectation values of products of system operators factorize,

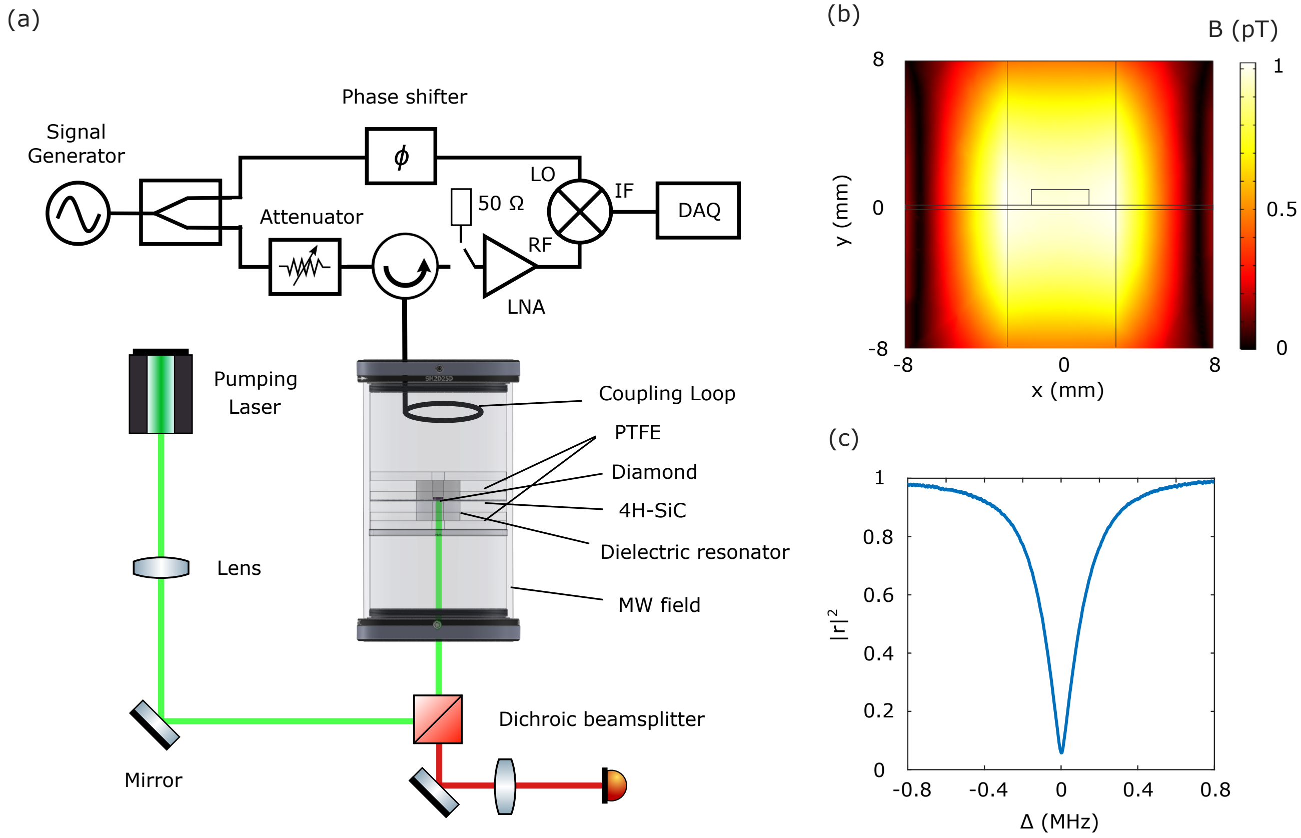

Supplementary Figure 1. (a). Experimental setup for cQED sensor. (b). COMSOL simulation for the dielectric resonator. Our dielectric resonator contains two cylinder with outer diameter: 0.669 inch; inner diameter: 0.236 inch; Thickness: 0.314 inch. (c). The cavity measurement with critical coupling. The loaded linewidth is 250 kHz, corresponding to an intrinsic linewidth of $\kappa_{c1} = 125$ kHz. The cavity center frequency is around 2.877 GHz.

the Bloch-Maxwell equations of motions for the cavity field, spin coherence, and polarization are [4]:

$$\dot{\alpha} = -\left(i\Delta + \frac{\kappa}{2}\right)\alpha - ig_{\rm s}\sum_j s_j + \sqrt{\kappa_{\rm c1}}\beta_{\rm in} \tag{2a}$$

$$\dot{s}_j = -\left(i\Delta_j + \frac{\gamma}{2}\right)s_j - ig_{\rm s}\mathcal{P}_j\alpha \tag{2b}$$

$$\dot{\mathcal{P}}_j = -\gamma_{\rm p}\left(\mathcal{P}_j - \bar{\mathcal{P}}\right) - 2ig_{\rm s}\left(s_j\alpha^* - s_j^*\alpha\right). \tag{2c}$$

Here α is the expectation value for the cavity field, $\mathcal{P}_j = p_{0j} - p_{1j}$ is the polarization—the population difference between the $m_{\rm s} = 0$ (p_{0j}) and $m_{\rm s} = 1$ (p_{1j}) populations, $\Delta = \omega_{\rm c} - \omega_{\rm d}$ is the cavity detuning and $\Delta_j = \omega_j - \omega_{\rm d}$ is the spin detuning for the jth spin. The polarization of the ensemble in the absence of the microwave drive is $\bar{\mathcal{P}}$. We relate $\gamma_{\rm p}$ and $\bar{\mathcal{P}}$ to the optical illumination intensity and properties of the NV optical polarization cycle in Sec. II D. We are interested in the steady-state solution,

$$\begin{aligned} \alpha &= \frac{\sqrt{\kappa_{\rm c1}}\beta_{\rm in} - ig_{\rm s}\sum_j s_j}{\kappa/2 + i\Delta} \\ s_j &= -i\mathcal{P}_j g_{\rm s}\alpha\frac{1}{\gamma/2 + i\Delta_j} \\ \mathcal{P}_j &= \bar{\mathcal{P}}_j - \frac{2ig_{\rm s}}{\gamma_{\rm p}}(\alpha^* s_j - \alpha s_j^*). \end{aligned} \tag{3}$$

We proceed to eliminate s_j and $\mathcal{P}_j$ to find the cavity field, from which the reflection coefficient, $r = -1 + \sqrt{\kappa_{\rm c1}}\alpha/\beta_{\rm in}$,

may be determined:

$$\alpha = \frac{\sqrt{\kappa_{c1}}\beta_{in}}{\kappa/2 + i\Delta} - g_s^2 \alpha \sum_j \frac{\mathcal{P}_j}{(\kappa/2 + i\Delta)(\gamma/2 + i\Delta_j)} \tag{4}$$

$$\Rightarrow \alpha = \frac{\sqrt{\kappa_{c1}}\beta_{in}}{i\Delta + \kappa/2 + g_s^2 \sum_j \frac{\mathcal{P}_j}{i\Delta_j + \gamma/2}} \tag{5}$$

$$\mathcal{P}_j = \bar{\mathcal{P}} - 2\frac{ig_s}{\gamma_p}\left[\frac{-ig_s|\alpha|^2\mathcal{P}_j}{i\Delta_j + \gamma/2} - \frac{ig_s|\alpha|^2\mathcal{P}_j}{-i\Delta_j + \gamma/2}\right]$$

$$= \bar{\mathcal{P}} - 2g_s^2|\alpha|^2 \frac{\gamma}{\gamma_p}\frac{1}{\gamma^2/4 + \Delta_j^2}\mathcal{P}_j$$

$$\Rightarrow \mathcal{P}_j = \frac{\bar{\mathcal{P}}}{1 + 2g_s^2|\alpha|^2 \frac{\gamma}{\gamma_p}\frac{1}{\Delta_j^2 + \gamma^2/4}}. \tag{6}$$

Eliminating $\mathcal{P}_j$ from the cavity field equation yields

$$\alpha = \frac{\sqrt{\kappa_{c1}}\beta_{in}}{i\Delta + \frac{\kappa}{2} + \bar{\mathcal{P}} g_s^2 \sum_j \frac{-i\Delta_j + \gamma/2}{\Delta_j^2 + \frac{\gamma^2}{4} + 2g_s^2|\alpha|^2\gamma/\gamma_p}}. \tag{7}$$

Next we define $\chi = \sqrt{1 + \frac{8g_s^2|\alpha|^2}{\gamma\gamma_p}}$ to parameterize the degree of nonlinearity in the system. The argument, $8g_s^2|\alpha|^2 \big/ \gamma_p\gamma$ is analagous to the standard saturation parameter: for $8g_s^2|\alpha|^2 \ll \gamma\gamma_p$ (equivalently, $\chi \sim 1$), the system is described by linear response. For an ensemble of $N \gg 1$ NV centers with $m_s = 1$ transition frequency distributed according to $P(\omega')$, we may take a continuum limit to find

$$\alpha = \frac{\sqrt{\kappa_{c1}}\beta_{in}}{i\Delta + \frac{\kappa}{2} + g^2 \int \frac{\gamma/2 - i(\omega' - \omega_d)}{(\omega - \omega')^2 + \chi^2\gamma^2/4} P(\omega') d\omega'}, \tag{8}$$

where we have introduced the collective coupling strength $g = \sqrt{\bar{\mathcal{P}} N} g_s$ between the ensemble and the cavity. Equation (8) represents the formal solution for the coupling between the dielectric resonator and the optically-polarized spin ensemble with inhomogeneous broadening. As discussed in the main text, both sides of the equation contain α, indicating that the energy oscillates between the cavity photon and the NV ensemble.

B. Ensemble distribution

The integral in the denominator of Supplementary Supplementary Eq. (8),

$$\mathcal{I} = \int \frac{\gamma/2 - i(\omega' - \omega_d)}{(\omega' - \omega_d)^2 + \chi^2\gamma^2/4} P(\omega')\, d\omega', \tag{9}$$

is the primary obstacle to a nonlinear semiclassical description of the inhomogeneous system. While it cannot be evaluated in general, it may be treated asymptotically in the relevant limit of small detuning and large inhomogeneity. The result of this analysis yields intuition for the parametric dependence of the signal on, e.g., inhomogeneous linewidth and optical polarization rate.

For an ensemble driven on resonance with the cavity ($\omega_d = \omega_c$), $\mathcal{I}$ may be rewritten as

$$\mathcal{I} \sim \int \left[\frac{\gamma}{2} - i(\omega' - \omega_c)\right] \frac{1}{(\omega' - \omega_c)^2 + \gamma^2\chi^2/4} P(\omega')$$

$$= \frac{2\pi}{\gamma\chi} \int \left[\frac{1}{\chi} - i\left(\frac{\omega' - \omega_c}{\gamma\chi/2}\right)\right] \frac{1}{\pi} \frac{1}{\left(\frac{\omega' - \omega_c}{\gamma\chi/2}\right)^2 + 1} P(\omega')\, d\omega'. \tag{10}$$

We label the standard Lorentz distribution in the integrand as f,

$$f(x) = \frac{1}{\pi}\frac{1}{x^2 + 1}, \tag{11}$$

and rewrite the spin inhomogeneous distribution in terms of a standardized lineshape function, h,

$$P(\omega') = \frac{2}{\Gamma} h\left(\frac{\omega_{\mathrm{s}} - \omega'}{\Gamma/2}\right). \tag{12}$$

Here Γ is the ensemble inhomogeneous linewidth and ω_{s} is the frequency at which the ensemble frequency distribution is peaked. The ensemble lineshape function, h, is maximal at zero argument, and has unit width and integral. The purpose of this manipulation is to recast the components of the integrand in terms of the standardized functions f and h, and dimensionful prefactors. Measuring frequency relative to ω_{s} ($\nu = \omega_{\mathrm{s}} - \omega'$, $\Delta_{\mathrm{s}} = \omega_{\mathrm{s}} - \omega_{\mathrm{c}}$), we have

$$\mathcal{I} = \frac{4\pi}{\Gamma\gamma\chi} \int \left[\frac{1}{\chi} + i\left(\frac{\Delta_{\mathrm{s}} + \nu}{\gamma\chi/2}\right)\right] f\left(\frac{\Delta_{\mathrm{s}} + \nu}{\gamma\chi/2}\right) h\left(\frac{\nu}{\Gamma/2}\right) \mathrm{d}\nu\,. \tag{13}$$

Our system operates in the regime $\Delta_{\mathrm{s}} \ll \gamma$. Noting $\chi \geq 1$, we expand Supplementary Supplementary Eq. (13) to linear order in $\Delta_{\mathrm{s}}/\gamma\chi$:

$$\begin{aligned}\mathcal{I} \sim \frac{4\pi}{\Gamma\gamma\chi} \Bigg\{ &\int \left(\frac{1}{\chi} + i\frac{\nu}{\gamma\chi/2}\right) f\left(\frac{\nu}{\gamma\chi/2}\right) h\left(\frac{\nu}{\Gamma/2}\right) \mathrm{d}\nu \\ &+ \frac{\Delta_{\mathrm{s}}}{\gamma\chi/2} \int \left[i f\left(\frac{\nu}{\gamma\chi/2}\right) + \left(\frac{1}{\chi} + i\frac{\nu}{\gamma\chi/2}\right) f'\left(\frac{\nu}{\gamma\chi/2}\right)\right] h\left(\frac{\nu}{\Gamma/2}\right) \mathrm{d}\nu \Bigg\}\,. \end{aligned} \tag{14}$$

To proceed, we examine the consequence of ensemble inhomogeneity, $\gamma \ll \Gamma$, on the above expression in the linear regime where $\chi \sim 1$ (and therefore $\gamma\chi \ll \Gamma$). In the region $\nu \ll \Gamma/2$ the inhomogeneous lineshape is necessarily approximately even about its peak in frequency. The contribution of terms that are odd within this approximation to the integrand of Supplementary Supplementary Eq. (14) will be suppressed by a small factor of $\gamma\chi/\Gamma$. Measuring frequency in units of $\gamma\chi/2$ (defining $y = 2\nu/\gamma\chi$), the integral is

$$\mathcal{I} \sim \frac{2\pi}{\Gamma\chi} \left\{ \underbrace{\int f(y) h\left(y\frac{\gamma\chi}{\Gamma}\right) \mathrm{d}y}_{A} + i\frac{\Delta_{\mathrm{s}}}{\gamma/2} \underbrace{\int [f(y) + y f'(y)] h\left(y\frac{\gamma\chi}{2}\right) \mathrm{d}y}_{B} \right\}. \tag{15}$$

By construction, A and B are of order 1. Recalling $\omega = \omega_{\mathrm{c}} \to \Delta = 0$, this expression may be substituted into Supplementary Supplementary Eq. (8) to yield

$$\alpha = \frac{\sqrt{\kappa_{\mathrm{c1}}}\beta_{\mathrm{in}}}{i\frac{4\pi g^2}{\gamma\Gamma\chi} B\Delta_{\mathrm{s}} + \frac{\kappa}{2} + \frac{2\pi g^2}{\Gamma\chi} A}. \tag{16}$$

In the limit of large microwave drive power such that $\gamma\chi \gg \Gamma$, one may simply neglect the ensemble inhomogeneity as a small correction and the standard homogeneous analysis may be applied.

1. *Gaussian distribution*

For a Gaussian inhomogeneous distribution,

$$P_{\mathrm{G}}(\omega) = \frac{1}{\sqrt{\pi}\Gamma/\eta} e^{-\frac{1}{2}\left(\frac{\omega - \omega_{\mathrm{s}}}{\Gamma/\eta}\right)^2}, \tag{17}$$

the integral from Supplementary Eq. (8) may be written in terms of the scaled complementary error function

$$\int \frac{\gamma/2 - i(\omega' - \omega_{\mathrm{d}})}{\gamma^2/4 + (\omega' - \omega_{\mathrm{d}})^2 + \gamma^2(\chi^2 - 1)/4} P_G(\omega') d\omega' = \int \frac{\chi/2 - i(\omega' - \omega_{\mathrm{d}})/\gamma + (1-\chi)/2}{\chi^2/4 + (\omega' - \omega_{\mathrm{d}})^2} P_G(\omega') d\omega' \tag{18}$$

$$\begin{aligned} &= \frac{\eta\sqrt{\pi}}{\Gamma} \left\{ \mathrm{erfcx}\left[\frac{\eta(\chi\gamma/2 + i\Delta_{\mathrm{d}})}{\sqrt{2}\Gamma}\right] + \frac{\gamma - \chi\gamma}{2\chi\gamma}\left(\mathrm{erfcx}\left[\frac{\eta(\chi\gamma/2 - i\Delta_{\mathrm{d}})}{\sqrt{2}\Gamma}\right] + \mathrm{erfcx}\left[\frac{\eta(\chi\gamma/2 + i\Delta_{\mathrm{d}})}{\sqrt{2}\Gamma}\right]\right)\right\} \\ &= \frac{\eta\sqrt{\pi}}{\Gamma} \left\{ \frac{\gamma + \chi\gamma}{2\chi\gamma} \mathrm{erfcx}\left[\frac{\eta(\chi\gamma/2 + i\Delta_{\mathrm{d}})}{\sqrt{2}\Gamma}\right] + \frac{\gamma - \chi\gamma}{2\chi\gamma} \mathrm{erfcx}\left[\frac{\eta(\chi\gamma/2 - i\Delta_{\mathrm{d}})}{\sqrt{2}\Gamma}\right]\right\}, \end{aligned} \tag{19}$$

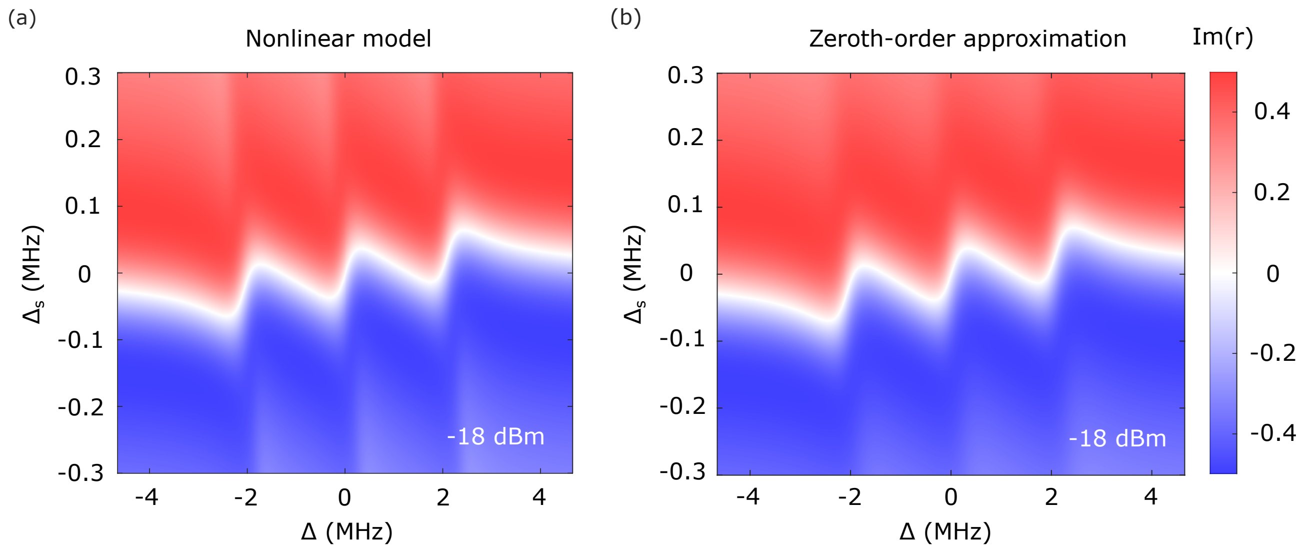

Supplementary Figure 2. Zeroth-order approximation of the model. (a). The imaginary part of the reflection coefficient determined by numerically solving the nonlinear model. (b). The zeroth-order approximation to the nonlinear model. The parameters used are described at the end of Sec. I.

with $\Delta_\mathrm{d} = \omega_\mathrm{s} - \omega_\mathrm{d}$. The factor $\eta = 2\sqrt{\ln 2}$ relates the Gaussian standard deviation to its full-width half-max. In the linear regime, this simplifies to

$$\frac{\eta\sqrt{\pi}}{\Gamma}\mathrm{erfcx}\left[\frac{\eta(\gamma/2 + i\Delta_\mathrm{d})}{\sqrt{2}\Gamma}\right]. \tag{20}$$

While asymptotic expressions for Supplementary Eq. (19) may be found, it is more convenient to parameterize the inhomogeneous distribution as Lorentzian because it yields a good fit to the measurements, and it allows this integral to be evaluated explicitly, providing an expression that interpolates cleanly between the weakly driven (inhomogeneous) and strongly driven (homogeneous) limits. As we have argued above, the exact details of the ensemble inhomogeneous distribution are not important at the sensor operating point.

2. *Lorentzian distribution*

For a Lorentzian-distributed ensemble,

$$P_\mathrm{L}(\omega) = \frac{1}{2\pi\Gamma}\frac{\Gamma^2}{(\omega - \omega_\mathrm{s})^2 + \Gamma^2/4}, \tag{21}$$

in the linear regime, the integral in the denominator of Supplementary Eq. (8) evaluates simply as

$$\begin{aligned}\int \frac{\gamma/2 - i(\omega' - \omega_\mathrm{d})}{\gamma^2/4 + (\omega' - \omega_\mathrm{d})^2} P_L(\omega')d\omega' &= \frac{(\gamma+\Gamma)/2 - i(\omega_\mathrm{s} - \omega_\mathrm{d})}{(\gamma+\Gamma)^2/4 + (\omega_\mathrm{s} - \omega_\mathrm{d})^2} \\ &= \frac{1}{(\gamma+\Gamma)/2 + i(\omega_\mathrm{s} - \omega_\mathrm{d})}.\end{aligned} \tag{22}$$

In the nonlinear regime, the integration yields a slightly more cumbersome expression,

$$\int \frac{\gamma/2 - i(\omega' - \omega_\mathrm{d})}{\gamma^2/4 + (\omega' - \omega_\mathrm{d})^2 + \gamma^2(\chi^2 - 1)/4} P_L(\omega')d\omega' = \frac{2\gamma + 2\Gamma/\chi - 4i\Delta_\mathrm{d}}{(\Gamma + \chi\gamma)^2 + 4\Delta_\mathrm{d}^2}. \tag{23}$$

At this point, we introduce an effective ensemble linewidth, $\Gamma_1 = \Gamma + \chi\gamma$ and collective coupling strength $g_\mathrm{eff} = g/\sqrt{\chi}$ to account for power broadening and quenching of the NV ensemble due to the microwave drive. With these definitions we have

$$\alpha = \frac{\sqrt{\kappa_\mathrm{c1}}\beta_\mathrm{in}}{i\Delta + \frac{\kappa}{2} + g_\mathrm{eff}^2 \frac{\Gamma_1/2 - i\chi\Delta_\mathrm{d}}{(\Gamma_1/2)^2 + \Delta_\mathrm{d}^2}}. \tag{24}$$

Recalling that the reflection coefficient is $r = -1 + \sqrt{\kappa_{c1}}\alpha/\beta_{\rm in}$, the sensing signal (evaluated at $\Delta_{\rm s} = \Delta = 0$, in this case, $\Delta_{\rm s} = \Delta_{\rm d}$) is

$$\begin{aligned} S &= f(\beta_{\rm in}) \left.\frac{\partial\, {\rm Im}[r]}{\partial \Delta_{\rm s}}\right|_{\Delta=\Delta_{\rm s}=0} \\ &\sim f(\beta_{\rm in}) \frac{\partial}{\partial \Delta_{\rm s}} {\rm Im}\left[\frac{\kappa_{c1}}{\frac{\kappa}{2} + \frac{2g_{\rm eff}^2}{\Gamma_1} - i\frac{2g_{\rm eff}^2}{\Gamma_1}\frac{\chi\Delta_{\rm s}}{\Gamma_1/2}}\right]\Bigg|_{\Delta_{\rm s}=0} \\ &\sim f(\beta_{\rm in}) \frac{4}{\Gamma_1}\frac{\kappa_{c1}}{\kappa}\frac{\chi C_\alpha}{(1+C_\alpha)^2}. \end{aligned} \tag{25}$$

In the final line we have introduced the effective cooperativity for the quenched system,

$$C_\alpha = \frac{4g_{\rm eff}^2}{\kappa\Gamma_1} = \frac{4g^2}{\chi\kappa(\Gamma+\chi\gamma)}, \tag{26}$$

as defined in the main text, and $f(\beta_{\rm in}) = \sqrt{\hbar\omega R}\beta_{\rm in}/L$, where R is the standard 50 Ω resistance.

To calculate the sensing signal one must determine the cavity occupancy $|\alpha|^2$ (or equivalently χ and hence C_α). One may multiply Supplementary Eq. (24) by its conjugate to determine a relationship between χ and $|\beta_{\rm in}|^2$:

$$|\alpha|^2 = \frac{\kappa_{c1}|\beta_{\rm in}|^2}{\left[\Delta - g^2\frac{\Delta_{\rm d}}{(\Gamma_1/2)^2+\Delta_{\rm d}^2}\right]^2 + \left[\frac{\kappa}{2} + g_{\rm eff}^2\frac{\Gamma_1/2}{(\Gamma_1/2)^2+\Delta_{\rm d}^2}\right]^2}. \tag{27}$$

While this is an octave equation for χ^2, it has only one positive real solution in the regime of cooperativity $C_0 \approx 1$, which is also the physical solution. When the system is tuned to resonance, the cavity occupancy is related to the MW drive power through

$$|\beta_{\rm in}|^2 = \frac{1}{\kappa_{c1}}\left(\frac{\kappa}{2} + \frac{1}{\chi}\frac{g^2}{\frac{\Gamma}{2}+\frac{\gamma\chi}{2}}\right)^2|\alpha|^2. \tag{28}$$

In the linear regime ($\chi \to 1$), we recover

$$|\beta_{\rm in}|^2 = \frac{1}{\kappa_{c1}}\left(\frac{\kappa}{2} + \frac{g^2}{\frac{\Gamma}{2}+\frac{\gamma}{2}}\right)^2|\alpha|^2. \tag{29}$$

C. Solution in the Nonlinear Regime

1. *Approximate treatment of cavity occupancy*

Equation (27) may be solved numerically, but it remains useful to seek an approximate analytic solution. We define an iterative procedure to approximate $|\alpha|^2$. At a given order we define

$$\chi^{(n)} = \sqrt{1 + 8\frac{\gamma}{\gamma_{\rm p}}\frac{g_{\rm s}^2|\alpha^{(n)}|^2}{\gamma^2}}, \tag{30}$$

and associated approximate ensemble linewidth, effective collective coupling and effective cooperativity,

$$\Gamma_1^{(n)} = \Gamma + \chi^{(n)}\gamma \qquad g_{\rm eff}^{(n)} = \frac{g}{\chi^{(n)}} \qquad C_\alpha^{(n)} = \frac{4{g_{\rm eff}^{(n)}}^2}{\kappa\Gamma_1^{(n)}}. \tag{31}$$

The next order is then found by iteratively evaluating Supplementary Eq. (27):

$$\left|\alpha^{(n+1)}\right|^2 = \frac{\kappa_{c1}|\beta_{\rm in}|^2}{\left[\Delta - g^2\frac{\Delta_{\rm d}}{\left(\Gamma_1^{(n)}/2\right)^2+\Delta_{\rm d}^2}\right]^2 + \left[\frac{\kappa}{2} + \left(g_{\rm eff}^{(n)}\right)^2\frac{\Gamma_1^{(n)}/2}{\left(\Gamma_1^{(n)}/2\right)^2+\Delta_{\rm d}^2}\right]^2}. \tag{32}$$

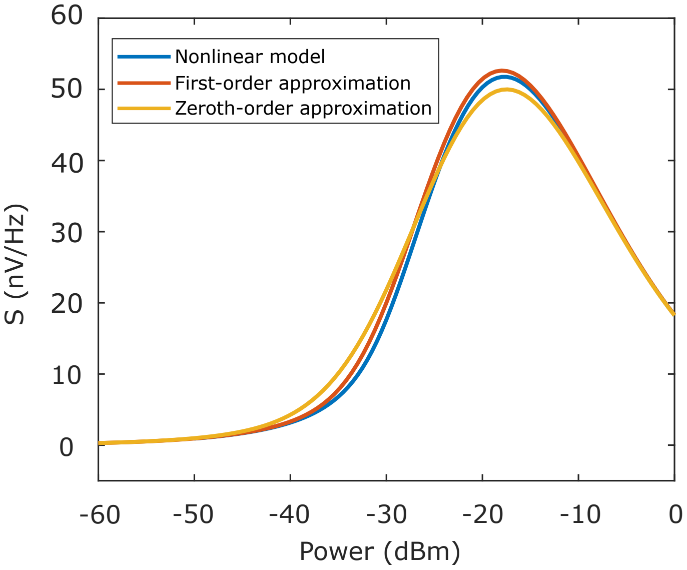

Supplementary Figure 3. Sensing signal predicted by the nonlinear model and approximate solutions for the parameter regime of our system. Blue: numerical solution for the nonlinear equation. Red: first-order approximation for the nonlinear model. Yellow: zeroth-order approximation for the nonlinear model. The parameters used are described at the end of Sec. I.

As a base case, we take the cavity occupancy to be its value in the presence of the MW drive but the absence of the NV ensemble,

$$\left|\alpha^{(0)}\right|^2 = 4\frac{\kappa_{c1}}{\kappa^2}|\beta_{\rm in}|^2. \tag{33}$$

The motivation for taking this saturated steady state as the base case is that the signal-maximizing microwave power is found to be near the onset of non-linearity approaching from above (see Sec. II C 3 for analysis of the linear and nonlinear regimes of the system). The "$n^{\rm th}$ order" approximation to the spectrum [sensing signal] may then be found by taking $\chi \to \chi^{(n)}$ in Supplementary Eq. (24) [Supplementary Eq. (69)].

As shown in Supplementary Figure 2, the leading-order approximation provides a reasonable estimate of the spectrum when the cooperativity, C_0, is not much larger than 1. The main departure between the numerically-determined result and the leading order approximation occurs in regions where the microwave drive frequency is resonant with the spin transition frequency. Here the zeroth-order approximation underestimates the cavity photon number when the driving frequency interacts with the NV spin ensemble.

We plot the sensing signal for numerically-determined cavity occupancy as well as the leading and first subleading approximations for the parameters characterizing our system in Supplementary Figure 3. The zeroth-order approximation deviates slightly from the full solution because it assumes $\alpha \sim 2\sqrt{\kappa_{c1}}\beta_{\rm in}/\kappa$, i.e. the complete absence of reflection. This assumption is not justified at the sensor operating point, where the influence of the NV ensemble results in a finite reflected signal. Taking subleading corrections approximately accounts for this, and yields good agreement with the numerically-determined behavior.

2. *Discussion of full solution*

Supplementary Figure 4 shows the magnitude of the reflection coefficient for the full numerical solution of Supplementary Eq. (27). Here, we choose the parameter sets $\kappa_{c1} = 2\pi \times 130$ kHz, $\kappa = 2\pi \times 260$ kHz, $\Gamma = 2\pi \times 330$ kHz, $\gamma_{\rm p} = 2\pi \times 2.23$ kHz, $\gamma = 2\pi \times 55$ kHz, $L = 0.53$, and $g = 2\pi \times 400$ kHz to illustrate the nonlinear model. Supplementary Figure 4(a) plots the one-dimensional linecut for $\Delta_{\rm s} = 0$ with different powers. In the linear regime, at weak microwave probe power, two polariton branches can be clearly observed. As the microwave power increases, the spin ensemble begins to quench, leading to a detuning-dependent decrease in the effective coupling strength. This quenching happens more easily when the spin frequency is in resonance with the detection frequency, resulting in three peaks along the linecut with microwave power ranging from -30 dBm to -20 dBm. Upon entering the strong-probe regime, a broadening effect is observed and the polariton branches collapse as the effective cooperativity is the increasingly suppressed. Ultimately, the ensemble is completely quenched when the power exceeds 0 dBm.

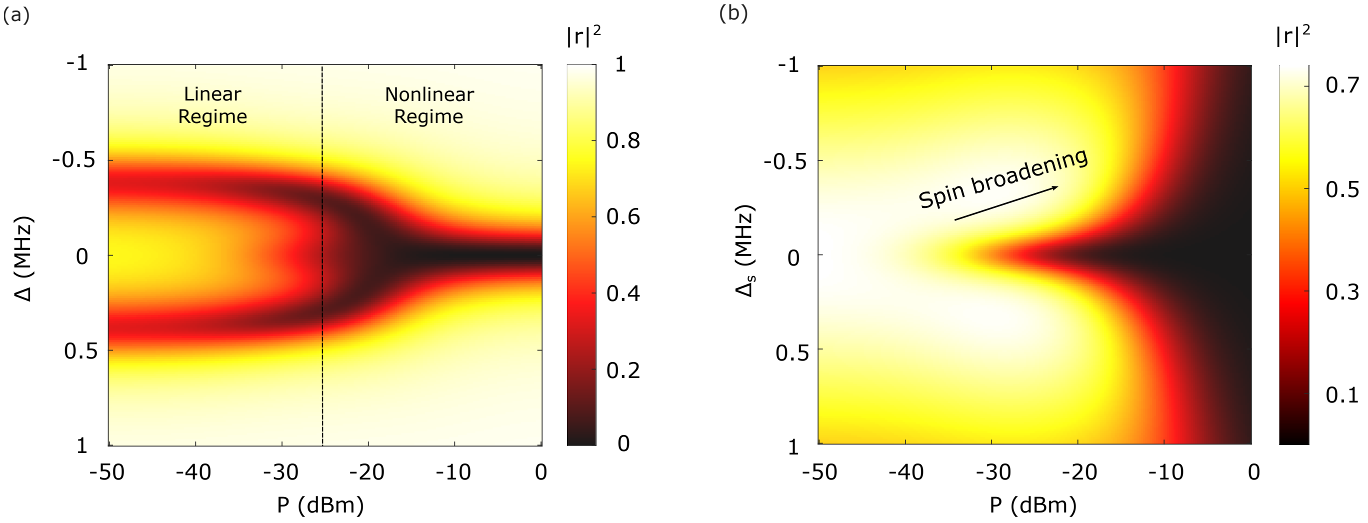

Supplementary Figure 4. (a,b) 1D linecut of the reflection power spectrum along Δ and Δ_s with different input microwave power with parameters: $\kappa_{c1} = 2\pi \times 130$ kHz, $\kappa = 2\pi \times 260$ kHz, $\Gamma = 2\pi \times 330$ kHz, $\gamma_p = 2\pi \times 2.23$ kHz, $\gamma = 2\pi \times 55$ kHz, $L = 0.53$, and $g = 2\pi \times 400$ kHz

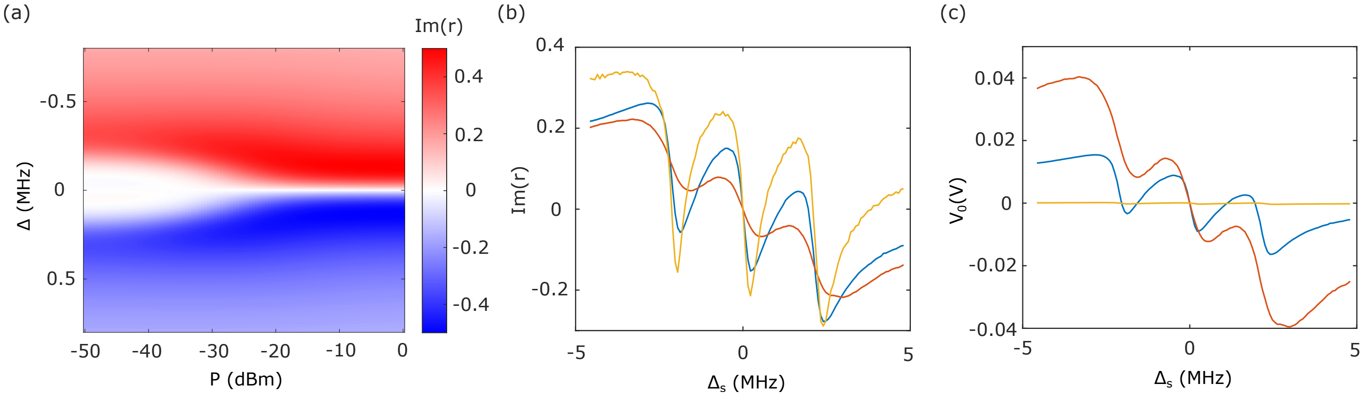

Supplementary Figure 5. (a) 1D linecut of the imaginary part of the reflection coefficient Im(r) for $\Delta_s = 0$ for different microwave power P. The parameters used are described at the end of Sec. I. (b) 1D linecut along $\Delta = 0$ for the voltage output. Yellow: -50 dBm, Blue: -18 dBm, Red: -8 dBm,. (c). 1D linecut along $\Delta = 0$ for the spectrum in Fig. 2b in the main text.

Supplementary Figure 4(b) shows the solution for the one-dimensional linecut $\Delta = 0$ with different powers. This figure highlights that the quenching effect initiates most easily at the spin resonant frequency. In the weak-probe regime, the linecut through $\omega_d = 0$ consistently yields high power. Upon entering the medium- or strong-power regime around -30 dBm of microwave power, the spin first quenches at the center when $\omega_s = \omega_d$. Additionally, we observe the microwave-induced broadening in Supplementary Figure 4(d), implying that the spin-quenching effect not only reduces the effective collective coupling strength but also expands the spin linewidth.

We also plot the imaginary part of the reflection coefficient in Supplementary Figure 5(a) for $\Delta = 0$ for different microwave powers. The linecut along $\Delta = 0$ in Fig. 2b of the main text is shown in Supplementary Figure 5(b). The nonlinear saturation effect can be clearly observed for different powers. We also plot the quadrature voltage in Supplementary Figure 5(c). This shows that the optimal signal (slope of the quadrature voltage) is found in the intermediate regime, although the largest slope for Im(r) is found at low power in Supplementary Figure 5(b).

3. Bistability Threshold

An interesting prediction of this model is for the possibility of bistable steady state solutions at certain microwave drive powers. Noting that our system is not sufficiently strongly coupled to realize this effect, we calculate the conditions required to realize bistability and input microwave photon flux range in which this effect would be observable.

This analysis is relevant to the projections we make in main text Sec. IV, where the optimal sensing operating point is found on the saturated branch for cooperative systems.

At the operating point $|\Delta_\mathrm{s}| \ll \gamma$, $\Delta = 0$, the intracavity photon number, $|\alpha|^2$, and incident microwave photon flux, $|\beta_\mathrm{in}|^2$, are related by Supplementary Eq. (28). Written in full, the relationship is

$$|\beta_\mathrm{in}|^2 = \frac{\kappa}{4}\frac{\kappa}{\kappa_\mathrm{c1}}\left[1 + \frac{4Ng_\mathrm{s}^2}{\kappa\sqrt{1+\frac{8g_\mathrm{s}^2}{\gamma^2}|\alpha|^2}\left(\Gamma + \gamma\sqrt{1+\frac{8g_\mathrm{s}^2}{\gamma^2}|\alpha|^2}\right)}\right]^2 |\alpha|^2. \tag{34}$$

This equation may, under certain conditions, be solved by multiple $|\alpha|^2$ for a given $|\beta_\mathrm{in}|$, corresponding to unsaturated or saturated configurations of the spin ensemble. To simplify the notation, we define

$$y = \frac{8g_\mathrm{s}^2}{\gamma^2}\frac{\gamma}{\gamma_\mathrm{p}}|\alpha|^2 \qquad x = \frac{32\kappa_\mathrm{c1}g_\mathrm{s}^2}{\gamma\gamma_\mathrm{p}\kappa^2}|\beta_\mathrm{in}|^2 \qquad C_0 = \frac{4g^2}{\kappa(\gamma+\Gamma)} \qquad \lambda = \frac{\Gamma}{\gamma}, \tag{35}$$

in which case

$$x = \left(1 + \frac{(1+\lambda)C_0}{1+y+\lambda\sqrt{1+y}}\right)^2 y. \tag{36}$$

Linearizing on the unsaturated branch, $y \ll 1$, for a cooperative system, $C_0 \gg 1$, yields the approximate unsaturated behavior:

$$x \sim C_0^2 y, \tag{37}$$

which is self consistent for $x \ll C_0^2$, or

$$|\beta_\mathrm{in}|^2 \ll \gamma_\mathrm{p}\frac{N}{2}\frac{\kappa}{\kappa_\mathrm{c1}}\frac{\gamma}{\Gamma+\gamma}\frac{g^2}{\kappa(\Gamma+\gamma)} \tag{38}$$

Linearizing on the saturated branch, $y \gg 1$, yields

$$x \sim y \tag{39}$$

provided

$$(1+\lambda)C_0 \ll 1 + y + \lambda\sqrt{1+y}. \tag{40}$$

This is self-consistent for either

$$x \gg (1+\lambda)C_0 \qquad\qquad x \gg C_0^2\left(\frac{1+\lambda}{\lambda}\right)^2. \tag{41}$$

As we are interested cooperative systems, $C_0 \gg 1$, the former limit is relevant, and we conclude that the saturated branch is stable for

$$|\beta_\mathrm{in}|^2 \gg \frac{N\gamma_\mathrm{p}}{8}\frac{\kappa}{\kappa_\mathrm{c1}}. \tag{42}$$

Examining these results, the saturated and unsaturated branches may both be stable for intermediate drive powers provided

$$\frac{4g^2}{\kappa(\Gamma+\gamma)}\frac{\gamma}{\gamma+\Gamma} \gg 1. \tag{43}$$

We plot the multiple solutions in Supplementary Figure 6. Within the critical power, there are more than one steady-state solution [5]. There are two stable states (yellow and blue) and an unstable solution (red). Operating on the unsaturated branch will give a better sensitivity based on this modeling, but optimal performance requires operation close to the edge of the bistable phase and potentially results in a nonlinear magnetic response. A full treatment of magnetometer operation deep in the bistable regime is needed to explore these possibilities.

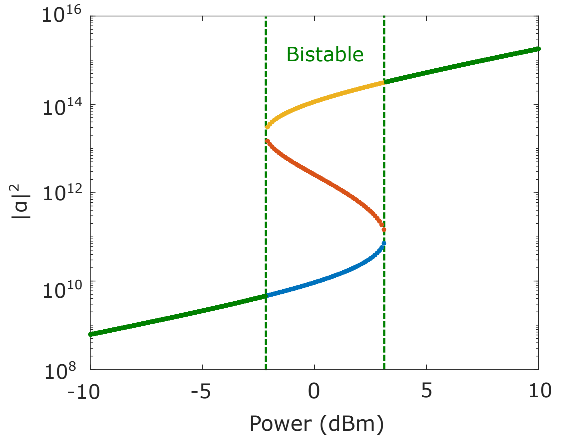

Supplementary Figure 6. Bistable steady state solutions. We plot the multiple solutions for cavity photon number $|\alpha|^2$ in the bistable regime within a critical power range. We use $g = 2$ MHz to make the plot instead of $g = 0.2$ MHz to observe the bistable regime.

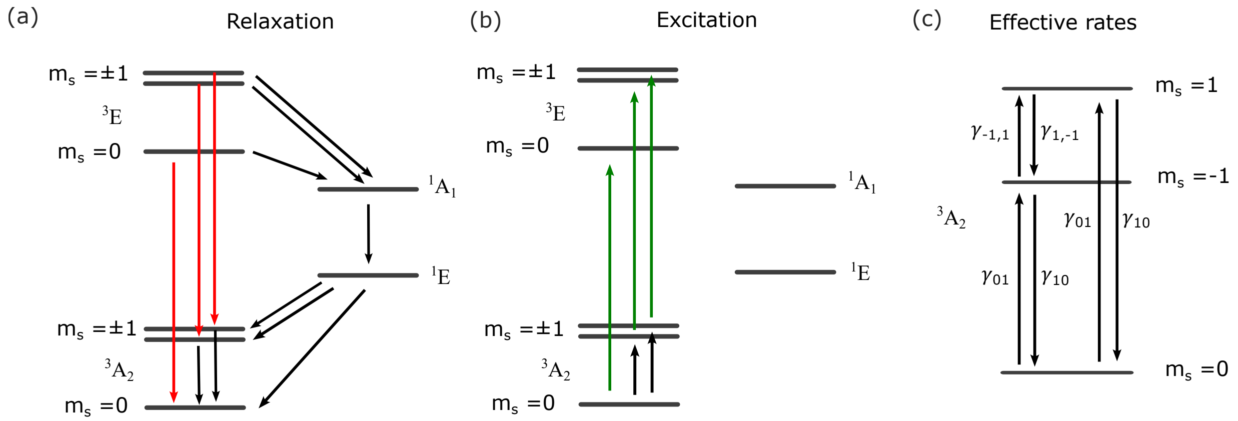

Supplementary Figure 7. Schematic of energy levels and rates involved in optical polarization cycle: (a) intrinsic relaxation rates and (b) excitation rates. The relevant rates are listed in Sec. II D with superscripts (subscripts) denoting the initial (final) state. Thermal excitation at optical frequencies is exponentially suppressed, so we neglect it. In panel (c) we illustrate the effective rates between the states in the $^3A_2$ subspace after eliminating the higher-energy states. These are tabulated in Supplementary Table 1 and used in the subsequent analysis.

D. Optical polarization cycle and generalized model

In the preceeding analysis we have asserted the existence of an equilibrium polarization $\bar{\mathcal{P}}$ and longitudinal relaxation rate γ_{p}, which presume to capture the effects of the optical cycle by which the ensemble is polarized. Additionally, we have apparently neglected the $m_s = -1$ state. While this treatment is typical, we have not yet justified it in terms of a more complete picture. In particular, we must relate these parameters to the optical excitation rate and intrinsic properties of the NV center. In this section, we provide such a systematic treatment of the full system's behavior under the optical polarization cycle. We review a rate equation model for the NV-polarization process in the absence of the microwave drive and then extend the semiclassical treatment of the driven NV-cavity ensemble to account for the relevant incoherent processes and the presence of the off-resonant $m_s = -1$ state. The model used in the main text and the model derived here are equivalent in the steady state.

1. *Effective rates for incoherent processes in the $^3A_2$ subspace*

The populations of the relevant NV states under optical pumping (but in the absence of a coherent microwave drive) may be described by a rate equation model [6], which is outlined here for reference (see, e.g., Jensen *et al.* [7] for a more detailed treatment). In the absence of an optical pump and at room temperature, the NVs relax to the $^3A_2$

Supplementary Table 1. Rates of thermal and optically-driven incoherent processes.

Process	Operator	Rate	Optical rate coefficient
$\|0\rangle \to \|-1\rangle$	$\|-1\rangle\langle 0\|$	$\gamma_{0,-1} \sim \gamma_{\rm th} + f_{0,-1}I$	$f_{0,-1} = f_{0,1} = r^{^3E,0}_{^1A_1} r^{^1E}_{^3A_2,\pm1} \approx 0.03$
$\|0\rangle \to \|0\rangle$	$\|0\rangle\langle 0\|$	$\gamma_{0,0} \sim f_{0,0}I$	$f_{0,0} = r^{^3E,0}_{^3A_2,0} + r^{^3E,0}_{^1A_1} r^{^1E}_{^3A_2,0} \approx 0.94$
$\|0\rangle \to \|1\rangle$	$\|1\rangle\langle 0\|$	$\gamma_{0,1} \sim \gamma_{\rm th} + f_{0,1}I$	$f_{0,1} = r^{^3E,0}_{^1A_1} r^{^1E}_{^3A_2,\pm1} \approx 0.03$
$\|1\rangle \to \|-1\rangle$	$\|-1\rangle\langle 1\|$	$\gamma_{1,-1} \sim f_{1,-1}I$	$f_{1,-1} = r^{^3E,\pm1}_{^1A_1} r^{^1E}_{^3A_2,\pm1} \approx 0.13$
$\|1\rangle \to \|0\rangle$	$\|0\rangle\langle 1\|$	$\gamma_{1,0} \sim \gamma_{\rm th} + f_{1,0}I$	$f_{1,0} = r^{^3E,\pm1}_{^1A_1} r^{^1E}_{^3A_2,0} \approx 0.32$
$\|1\rangle \to \|1\rangle$	$\|1\rangle\langle 1\|$	$\gamma_{1,1} \sim f_{1,1}I$	$f_{1,1} = r^{^3E,\pm1}_{^3A_2,\pm1} + r^{^3E,\pm1}_{^1A_1} r^{^1E}_{^3A_2,\pm1} \approx 0.55$
$\|-1\rangle \to \|-1\rangle$	$\|-1\rangle\langle -1\|$	$\gamma_{-1,-1} \sim f_{-1,-1}I$	$f_{-1,-1} = f_{1,1} = r^{^3E,\pm1}_{^3A_2,\pm1} + r^{^3E,\pm1}_{^1A_1} r^{^1E}_{^3A_2,\pm1} \approx 0.55$
$\|-1\rangle \to \|0\rangle$	$\|0\rangle\langle -1\|$	$\gamma_{-1,0} \sim \gamma_{\rm th} + f_{-1,0}I$	$f_{-1,0} = f_{1,0} = r^{^3E,\pm1}_{^1A_1} r^{^1E}_{^3A_2,0} \approx 0.32$
$\|-1\rangle \to \|1\rangle$	$\|1\rangle\langle -1\|$	$\gamma_{-1,1} \sim f_{-1,1}I$	$f_{-1,1} = f_{1,-1} = r^{^3E,\pm1}_{^1A_1} r^{^1E}_{^3A_2,\pm1} \approx 0.13$

subspace, where the populations will thermalize to equal occupancy through transitions between states of $|\Delta m_{\rm s}| = 1$ with rate $\gamma_{\rm th} = 1/2T_1 \approx 90\ {\rm s}^{-1}$ assuming $T_1 \approx 5.6$ ms [8]. Polarization within this subspace may be generated by optical pumping: after spin-conserving optical excitation to the ^3E manifold, a spin-selective inter-system crossings preferentially allows population from the $^3E, m_{\rm s} = \pm1$ states to relax through intermediate singlet states, $^1A_1$ and ^1E, to the $^3A_2$ manifold, where it may end in the $^3A_2, m_{\rm s} = 0$ state [9]. This process is depicted in Supplementary Figure 7 a,b. The relaxation rates directly from the ^3E manifold and through the spin-selective inter-system crossing have been measured by, e.g., Gupta *et al.* [10] to be

- Spin-conserving m-independent relaxation from ^3E to $^3A_2$: $\Gamma^{^3E}_{^3A_2} \approx 66\ \mu{\rm s}^{-1}$
- Spin-selective inter-system crossing from ^3E to the singlet manifold: $\Gamma^{^3E,0}_{^1A_1} \approx 11\ \mu{\rm s}^{-1}$ and $\Gamma^{^3E,\pm1}_{^1A_1} \approx 92\ \mu{\rm s}^{-1}$
- Spin-selective inter-system crossing from ^1E to the $^3A_2$ manifold: $\Gamma^{^1E}_{^3A_2,0} \approx 4.9\ \mu{\rm s}^{-1}$ and $\Gamma^{^1E}_{^3A_2,\pm1} \approx 2.0\ \mu{\rm s}^{-1}$

Relaxation from vibrational excited states in ^3E and within the singlet subspace occurs much faster than the inter-system crossings and direct relaxation from ^3E to $^3A_2$ [11].

For optical excitation rate I much smaller than the aggregate relaxation processes, the vast majority of the NV population will remain in the $^3A_2$ subspace. In this case, one may determine effective rates between states within the $^3A_2$ manifold using the branching ratios of the post-optical-excitation relaxation pathways,

$$\begin{aligned}
r^{^3E,0}_{^3A_2,0} &= \frac{\Gamma^{^3E}_{^3A_2}}{\Gamma^{^3E}_{^3A_2} + \Gamma^{^3E,0}_{^1A_1}} \approx 0.86 & r^{^3E,0}_{^1A_1} &= \frac{\Gamma^{^3E,0}_{^1A_1}}{\Gamma^{^3E}_{^3A_2} + \Gamma^{^3E,0}_{^1A_1}} = 1 - r_{^3E,0\to{}^3A_2,0} \approx 0.14 \\
r^{^3E,\pm1}_{^3A_2,\pm1} &= \frac{\Gamma^{^3E}_{^3A_2}}{\Gamma^{^3E}_{^3A_2} + \Gamma^{^3E\pm1}_{^1A_1}} \approx 0.42 & r^{^3E,\pm1}_{^1A_1} &= \frac{\Gamma^{^3E,\pm1}_{^1A_1}}{\Gamma^{^3E}_{^3A_2} + \Gamma^{^3E,\pm1}_{^1A_1}} = 1 - r^{^3E,\pm1}_{^3A_2,\pm1} \approx 0.58 \\
r^{^1A_1}_{^1E} &= 1 \\
r^{^1E}_{^3A_2,0} &= \frac{\Gamma^{^1E}_{^3A_2,0}}{\Gamma^{^1E}_{^3A_2,0} + 2\Gamma^{^1E}_{^3A_2,\pm1}} \approx 0.55 & r^{^1E}_{^3A_2,\pm1} &= \frac{\Gamma^{^1E}_{^3A_2,\pm1}}{\Gamma^{^1E}_{^3A_2,0} + 2\Gamma^{^1E}_{^3A_2,\pm1}} = \frac{1}{2}\left(1 - r^{^1E}_{^3A_2,0}\right) \approx 0.22,
\end{aligned} \tag{44}$$

and the optical excitation rate. These effective rates are tabulated in Supplementary Table 1 and the effective rate equations are

$$\frac{\rm d}{{\rm d}t}\begin{pmatrix} p_{0j} \\ p_{-1j} \\ p_{1j} \end{pmatrix} = \begin{pmatrix} -2\gamma_{\rm th} - 2f_{0,1}I & \gamma_{\rm th} + If_{1,0} & \gamma_{\rm th} + f_{1,0}I \\ \gamma_{\rm th} + f_{0,1}I & -\gamma_{\rm th} - (f_{1,0} + f_{1,1})I & f_{1,1}I \\ \gamma_{\rm th} + f_{0,1}I & f_{1,1}I & -\gamma_{\rm th} - (f_{1,0} + f_{1,1})I \end{pmatrix}\begin{pmatrix} p_{0j} \\ p_{-1j} \\ p_{1j} \end{pmatrix}. \tag{45}$$

Note that relaxation within the singlet subspace does not have any associated branching and this step is suppressed

in our notation. In the steady state, the populations are

$$\begin{pmatrix} p_{0j} \\ p_{-1j} \\ p_{1j} \end{pmatrix} \propto \begin{pmatrix} \gamma_{\text{th}} + f_{1,0} I \\ \gamma_{\text{th}} + f_{0,1} I \\ \gamma_{\text{th}} + f_{0,1} I \end{pmatrix}. \tag{46}$$

The NV centers will be thermally depolarized for

$$I \ll \frac{\gamma_{\text{th}}}{f_{1,0}} \approx 280 \text{ s}^{-1}. \tag{47}$$

Above this scale the ensemble becomes increasingly optically polarized and asymptotically approaches the limiting populations

$$P_{^3A_2,0} \sim \frac{f_{1,0}}{f_{1,0} + 2f_{0,1}} \approx 0.84 \tag{48}$$

$$P_{^3A_2,\pm 1} \sim \frac{f_{0,1}}{f_{1,0} + 2f_{0,1}} \approx 0.08, \tag{49}$$

for

$$I \gg \frac{\gamma_{\text{th}}}{f_{0,1}} \approx 3 \text{ ms}^{-1}. \tag{50}$$

The associated laser fluence is determined by the 532 nm photon energy ($\approx 3.7 \times 10^{-19}$ J) and the optical excitation cross section, for which values between $\sigma_{^3A_2 \to ^3E} = 3 \times 10^{-17}$ cm^2 and $\sigma_{^3A_2 \to ^3E} = 9.5 \times 10^{-17}$ cm^2 are reported [11, 12]. For comparison, an 8 W laser power focused to a 3×3 mm^2 spot size corresponds between $I \approx 7$ ms^{-1} and $I \approx 24.5$ ms^{-1}. Our fitting result shows $I = 18.8$ ms^{-1}, within the reasonable range in the literature.

2. *Generalized Maxwell-Bloch equations*

The rates of these incoherent processes may also be included in the equations of motion for the driven system operators (see also Dréau *et al.* [13] and Wang *et al.* [14]). Including the coherent drive, the Hamiltonian may be written as [1, 2]:

$$\begin{aligned} H = {} & \omega_c a^\dagger a + \sum \omega_j \sigma_j^\dagger \sigma_j + \sum g_s (a^\dagger \sigma_j + a \sigma_j^\dagger) + i\sqrt{\kappa_{c1}} \beta_{\text{in}} (a^\dagger e^{-i\omega_d t} - a e^{i\omega_d t}) \\ & + \sum \omega_{-1,j} \sigma_{-1,j}^\dagger \sigma_{-1,j} + \sum g_s (a^\dagger \sigma_{-1,j} + a \sigma_{-1,j}^\dagger). \end{aligned} \tag{51}$$

Here $\sigma_j = |0\rangle\langle +1|_j$ as before and we have introduced the transition operator for $m_s = -1$ state, $\sigma_{-1,j} = |0\rangle\langle -1|_j$. In the rotating frame defined by $|\varphi\rangle \to e^{-i\omega_d t (a^\dagger a + \sum \sigma_j^\dagger \sigma_j + \sum \sigma_{-1,j}^\dagger \sigma_{-1,j})} |\varphi\rangle$, the Hamiltonian transforms to

$$H = \Delta_c a^\dagger a + \sum \Delta_j \sigma_j^\dagger \sigma_j + \sum g_s (a^\dagger \sigma_j + a \sigma_j^\dagger) + i\sqrt{\kappa_{c1}} \beta_{\text{in}} (a^\dagger - a) + \sum \Delta_{-1,j} \sigma_{-1,j}^\dagger \sigma_{-1,j} + \sum g_s (a^\dagger \sigma_{-1,j} + a \sigma_{-1,j}^\dagger). \tag{52}$$

The Maxwell-Bloch equations may be found by evaluating

$$\dot{A} = i[H, A] + \sum_\lambda \gamma_\lambda \left(L_\lambda^\dagger A L_\lambda - \frac{1}{2} \left\{ L_\lambda^\dagger L_\lambda, A \right\} \right), \tag{53}$$

for system operator A and taking the semiclassical limit. Here λ sums over the incoherent processes with rates and jump operators tabulated in Supplementary Table 1. Of course there may also be additional transverse relaxation mechanisms; we account for this possibility in the definition of the total decoherence rate for the spin coherence operators' equations of motion.

The $\text{m}_\text{s} = 0$ to $\text{m}_\text{s} = -1$ transition is far off resonance from the microwave drive that sustains the cavity field. The associated coherence, $s_{j,-1} = \langle \sigma_{-1,j} \rangle$, obeys semiclassical equation of motion

$$\dot{s}_{j,-1} = -[\gamma_{-1}/2 + i\Delta_j] s_{j,-1} + i g_s (p_{-1,j} - p_{0,j}) \alpha, \tag{54}$$

and decays to $s_{j,-1} \sim 0$ at this large detuning. Here $\gamma_{-1}/2$ is the total transverse relaxation rate for the transition. The $m_s = 1$ to $m_s = -1$ transition coherence operator is also far off resonance and may be neglected in the steady state.

The remaining steady-state semiclassical Maxwell-Bloch equations for the cavity field, $m_s = 1$ spin coherence, and populations are [4]

$$\dot{\alpha} = 0 = (-\kappa/2 + i\Delta)\alpha + \sqrt{\kappa_{c1}}\beta_{in} - ig_s \sum s_j \tag{55a}$$
$$\dot{s_j} = 0 = -[\gamma/2 + i\Delta_j]s_j + ig_s(p_{1j} - p_{0j})\alpha \tag{55b}$$
$$\dot{p}_{1j} = 0 = -(\gamma_{th} + f_{1,0}I)p_{1j} + (\gamma_{th} + f_{0,1}I)p_{0j} + f_{1,-1}Ip_{-1j} - f_{1,-1}Ip_{1j} + ig_s(\alpha s_j^* - \alpha^* s_j) \tag{55c}$$
$$\dot{p}_{-1j} = 0 = -(\gamma_{th} + f_{1,0}I)p_{-1j} - f_{1,-1}Ip_{-1j} + f_{1,-1}Ip_{1j} + (\gamma_{th} + f_{0,1}I)p_{0j} \tag{55d}$$
$$\dot{p}_{0j} = 0 = (\gamma_{th} + f_{1,0}I)(p_{1j} + p_{-1j}) - 2(\gamma_{th} + f_{0,1}I)p_{0j} - ig_s(\alpha s_j^* - \alpha^* s_j). \tag{55e}$$

The $m_s = 0 \to m_s = 1$ dephasing rate is

$$\gamma = 3\gamma_{th} + (f_{0,0} + 2f_{0,1} + f_{1,0} + f_{1,1} + f_{1,-1})I + \gamma_0$$
$$\approx 3\gamma_{th} + 2I + \gamma_0, \tag{56}$$

where γ_0 accounts for addition dephasing induced by, e.g., ^{13}C nuclear spin hyperfine interaction [15].

To work with more compact expressions, we restore the effective rates between the $^3A_2$ states, $\gamma_{\mu,\nu}$, defined in Supplementary Table 1. Noting that the overall population is conserved, $1 = p_{0j} + p_{1j} + p_{-1j}$, the Maxwell-Bloch equations for the population may be used to eliminate $p_{1j} - p_{0j}$ from Supplementary Eq. (55b):

$$\gamma_{1,0}p_{1j} + \gamma_{-1,0}p_{-1j} = (\gamma_{0,1} + \gamma_{0,-1})(1 - p_{1j} - p_{-1j}) + ig_s(\alpha s_j^* - \alpha^* s_j) \tag{57a}$$
$$(\gamma_{-1,0} + \gamma_{-1,1})p_{-1j} = \gamma_{1,-1}p_{1j} + \gamma_{0,-1}(1 - p_{1j} - p_{-1j}). \tag{57b}$$

Next we note $\gamma_{1,0} = \gamma_{-1,0}$ and $\gamma_{0,-1} = \gamma_{0,1}$ to simplify Supplementary Eq. (57a), yielding

$$p_{-1j} + p_{1j} = \frac{2\gamma_{0,1} + ig_s(\alpha s_j^* - \alpha^* s_j)}{2\gamma_{0,1} + \gamma_{1,0}}, \tag{58}$$

and

$$p_{1j} = \frac{\gamma_{0,1}}{2\gamma_{0,1} + \gamma_{1,0}} + \frac{\gamma_{1,0} + \gamma_{0,1} + \gamma_{1,-1}}{(2\gamma_{0,1} + \gamma_{1,0})(\gamma_{1,0} + 2\gamma_{1,-1})} \cdot ig_s(\alpha s_j^* - \alpha^* s_j). \tag{59}$$

The steady-state polarization is therefore,

$$\mathcal{P}_j \equiv p_{0j} - p_{1j} = \frac{\gamma_{1,0} - \gamma_{0,1}}{2\gamma_{0,1} + \gamma_{1,0}} - \frac{2\gamma_{1,0} + \gamma_{0,1} + 3\gamma_{1,-1}}{(2\gamma_{0,1} + \gamma_{1,0})(\gamma_{1,0} + 2\gamma_{1,-1})} \cdot ig_s(\alpha s_j^* - \alpha^* s_j). \tag{60}$$

Comparing to Supplementary Eq. (3), we can now identify the equilibrium polarization and effective optical polarization rate for

$$\bar{\mathcal{P}} = \frac{\gamma_{1,0} - \gamma_{0,1}}{2\gamma_{0,1} + \gamma_{1,0}} \tag{61}$$
$$\gamma_p = \frac{2(2\gamma_{0,1} + \gamma_{1,0})(\gamma_{1,0} + 2\gamma_{1,-1})}{2\gamma_{1,0} + \gamma_{0,1} + 3\gamma_{1,-1}}. \tag{62}$$

With this identification, the effective two-level model used in our analysis has the same steady-state behavior as the model presented here for the full $^3A_2$ subspace.

Using the numbers in Supplementary Table 1, we have:

$$\bar{\mathcal{P}} = \frac{0.29I}{3\gamma_{th} + 0.38I} \tag{63}$$
$$\gamma_p = \frac{2(3\gamma_{th} + 0.38I)(\gamma_{th} + 0.58I)}{3\gamma_{th} + 1.08I}. \tag{64}$$

III. NOISE ANALYSIS

A. Johnson-Nyquist limit

As discussed in the main text, the Johnson-Nyquist limit for the voltage measurement is $\sqrt{4k_BTR_s\Delta f}$. Here k_B is the Boltzmann constant. T is the cavity's temperature, R_s is the equivalent resistance of the composite cavity, and Δf

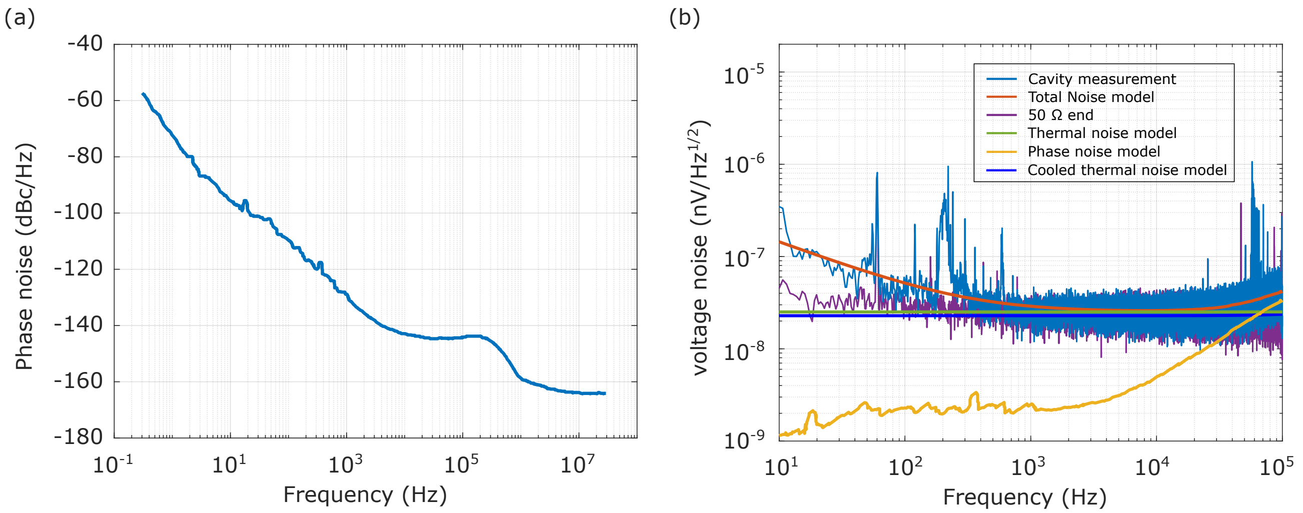

Supplementary Figure 8. (a). SSB phase noise for Rohde & Schwarz SMA100B signal generator. Data is from the specification from the Rohde & Schwarz website [16]. (b). Voltage noise for cavity measurement (blue), noise floor set by amplifier, mixer, and readout electronics measured by a 50 Ω end (purple), total noise model (red), thermal noise model (green), phase noise model (yellow), cooled thermal noise model (Dark blue).

is the single-sided measurement bandwidth. Assuming the impedence of the cavity matches the termination resistance $R = 50\ \Omega$, this produces a noise voltage $\mathcal{L}_{th} = \sqrt{k_B T R \Delta f}$ at the measurement device. The Johnson-Nyquist limit for the magnetic field sensitivity is given by:

$$\eta_{\rm JN} = \frac{\sqrt{k_B T R \Delta f}/F_0}{AS}. \tag{65}$$

Here $S = dQ_0/d\omega_{\rm s}$ is the signal measured by the mixer with Q_0 the quadrature voltage; A is the prefactor for different measuring systems, e.g. $A = \gamma \sin\theta$ for a magnetometer with $\gamma = 28$ GHz/T the gyromagnetic ratio and θ the angle between the applied field and NV axis; $F_0 = F_1 \cdot F_2 = 2$, where $F_1 = \sqrt{2}$ if the phase of the signal is assumed to be known; and $F_2 = \sqrt{2}$ assuming all signal is isolated into the quadrature channel of the mixer.

B. Phase noise

Besides the Johnson-Nyquist noise, we also experience phase noise induced by the signal generator. The signal from the signal generator is not perfect; it doesn't have a single frequency. We can express the microwave power as $\mathcal{L}_p(f) = 2\pi\delta(f) + \mathcal{L}_A(f) + \mathcal{L}_\varphi^{\rm osc}(f)$, where $\mathcal{L}_\varphi^{\rm osc}(f)$ represents the single sideband (SSB) phase noise at an offset frequency f from the carrier, and $\mathcal{L}_A(f)$ represents the amplitude noise at an offset frequency f from the carrier. We are considering the case where all signals are isolated to the quadrature channel. In this case, the phase noise is [17, 18]:

$$\mathcal{L}_\varphi(f) = \mathcal{L}_\varphi^{\rm osc}(f) |V_R \left[{\rm Re}(r) + i{\rm Im}(r) \right]|^2. \tag{66}$$

We ignore the amplitude noise for the following reasons: (1). The signal generator we use has a much lower amplitude noise $\mathcal{L}_A(f)$ compared to the phase noise $\mathcal{L}_\varphi^{\rm osc}(f)$. (2). We isolate the signal in the quadrature channel, and the system is much more sensitive to phase noise than amplitude noise. Supplementary Figure 8(a) illustrates the phase noise of the signal generator used in the experiment (Rohde & Schwarz SMA100B with ultra-low phase noise module). The phase noise spectrum shows lower noise at high frequencies, and both the quadrature and in-phase channels exhibit the strongest signal at $f = \kappa/2$. In our cavity design, $\kappa/2 \sim 130$ kHz with critical coupling, which explains the observed voltage noise at high frequencies.

In the phase noise analysis, we only consider the first-order voltage noise induced by the signal generator's phase noise. We omit the second-order term $\mathcal{L}_\varphi^{\rm sec}(f) = \mathcal{L}_\varphi^{\rm osc}(f_1) \cdot \mathcal{L}_\varphi^{\rm osc}(f_2) Q_0^2|_{f=|f_1-f_2|}$ because, in our case, the signal generator phase noise is sufficiently good, making the second-order term much smaller than the first-order term. Based on Supplementary Eq. (66), we find the phase noise depends on two factors: (1). Signal generator phase noise. (2). The quadrature RMS voltage from the cavity output port.

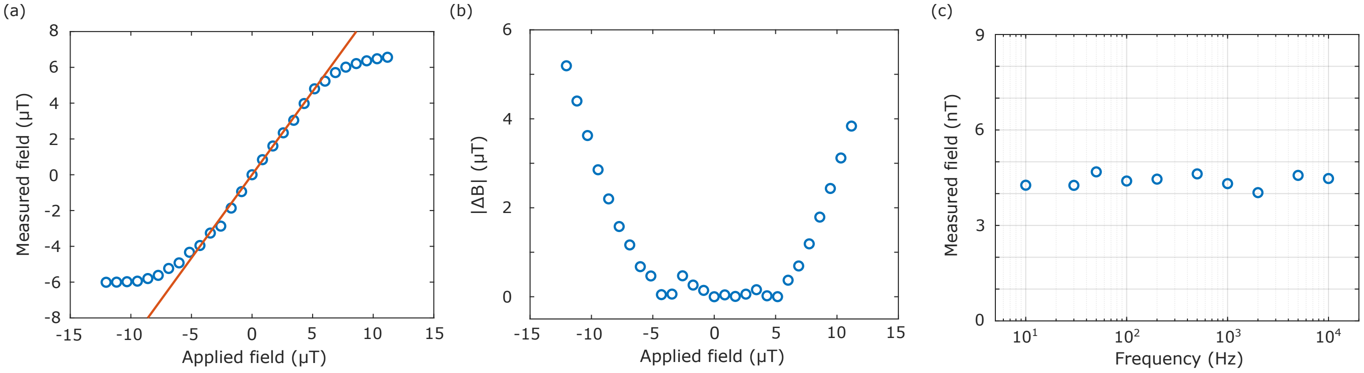

Supplementary Figure 9. (a). Dynamical range measurement for the cQED sensor. The sensor response is linear with applied magnetic field in a range of $\pm 5\ \mu T$. (b). Difference $|\Delta B|$ between the measurement and applied fields. (c). Sensor's response for different frequencies. The sensor's response for different frequencies varies within 6% in a range of 10 Hz to 10 kHz. This small difference may be attributed to (1) The magnetic field response for the materials and shield (2) Microwave delivery difference for different frequencies.

C. Other noise sources

The dominant noise sources at high frequencies include Johnson-Nyquist noise, phase noise, and noise figure (0.8 dB) for the amplifier. There is also noise present at low frequencies, which comprises (1) flicker noise and other high-order noise sources and (2) noise originating from the loop and cavity oscillation. Mechanical movement in the coupling loops leads to variations in the coupling strength with the cavity, thus gradually altering the cavity frequency. This results in substantial noise at low frequencies. In the experiments, these noise sources always remain below 1 kHz. To mitigate these noise sources, a lock-in amplifier can be used to upmodulate the detection to higher frequencies, thereby avoiding these low-frequency noise components.

Supplementary Figure 8(b) shows the noise spectrum and theory plots with models shown in the previous sections. First, the measurement shows the thermal noise we measured with a gain of 36.5 dB is 25.1 nV/$\sqrt{\text{Hz}}$, which consists of a 0.8 dB noise figure for the amplifier. Then using the combination of the nonlinear model and the mode cooling model, we plot the cooled thermal noise model with blue line. Including our phase noise model generated by Supplementary Eq. (66) with an input power of $P = -22$ dBm and the signal generator phase noise plotted in Supplementary Figure 8(a), we plot the phase noise for the magnetometer in Supplementary Figure 8(b). Finally, we added the flicker noise for the low-frequency bands with $\mathcal{L}^{1/2} = 450$ nV/$\sqrt{f}$.

D. Model for steady-state cooling in the nonlinear regime

We use a perturbation model for the fitting of the steady-state cooling. The linear theory gives the cavity output power spectrum as [19]:

$$N_P(\omega) = n_T + (n_{\mathrm{c}} - n_T)\, C, \tag{67}$$

where $n_T = [\exp(\hbar\omega/k_B T) - 1]^{-1}$ is the room temperature photon number and the effective 'cold spin bath" photon number n_{c}. The definition of C is:

$$C = \frac{\kappa_{\mathrm{c}} \Gamma_1 g_{\mathrm{eff}}^2}{\left(\frac{\Gamma_1^2}{4} + \Delta^2\right)\left[\frac{\kappa^2}{4} + \Delta_{\mathrm{s}}^2\right] + g_{\mathrm{eff}}^2 [\Gamma_1 \frac{\kappa}{2} - 2\Delta\Delta_{\mathrm{s}}] + g_{\mathrm{eff}}^4}. \tag{68}$$

In the nonlinear model with $\Delta = \Delta_{\mathrm{s}} = 0$, we approximatively treat the system as a linear system with $g_{\mathrm{eff}} = g/\sqrt{\chi}$ and $\Gamma_1 = \Gamma + \chi\gamma$. The deviation between this perturbation model and the strict model may come from two parts: (1). The above approximation is valid only when $\Delta_{\mathrm{s}} \ll \chi\Gamma_1/2$. When we are in the nonlinear regime, the coupling strength is always smaller than the cavity linewidth, meaning that we are no longer in the strong-coupling regime with a strong drive. Both the operation point and the best mode cooling spot happen together at $\Delta_{\mathrm{s}} = 0$. Therefore this approximation can be valid. (2). In the nonlinear model shown in the main text, the nonlinear term χ is dependent on $|\alpha|$ and implicitly cavity input β_{in}. Here we make an zeroth-order approximation $\alpha \sim \sqrt{\kappa_{\mathrm{c1}}}\beta_{\mathrm{in}}$ and this

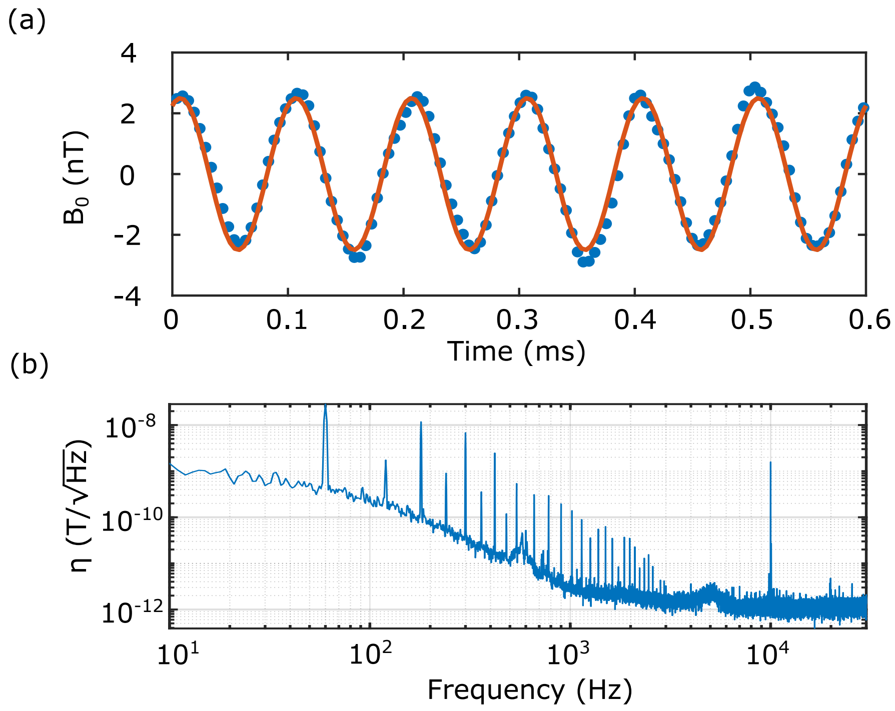

Supplementary Figure 10. (a). Time domain data for the sensor. A sinusoidal magnetic field with $B_{\rm pp} = 5$ nT generated by a functional generator is applied to the sensor. The low-frequency drifts are due to the low-frequency noises. (b). The sensitivity plot for the time domain data shown in (a).

approximation is valid around $\Delta_{\rm s} = 0$. We point out that this model uses the combination of the linear model with the nonlinear equivalent parameters. Future work should start from the input-output theory and cavity microwave photon distribution [19].

IV. SENSOR DETAILS

A. Dynamic range

Dynamic range is also an important parameter for a sensor. Supplementary Figure 9(a) shows the measurement for the applied magnetic field and the corresponding response of the cQED sensor. We plot the linear response with the red line as a comparison. We also plot the residual between these measurements and the linear response in Supplementary Figure 9(b). The linear region shows that the dynamical range for the cQED sensor is around 10 μT. The ratio between the dynamical range and the minimum detectable field with 1 Hz measurement bandwidth is 1.5×10^7.

B. Sensor response frequency range

In the main text, we show the broadband magnetic field sensitivity in Fig. 4(a) and describe the DC magnetic field calibration process in Methods. Here we demonstrate the sensor's response for different frequencies. In this measurement, we use a 2-inch lens tube with an iris as a microwave shield to get the intrinsic magnetic field sensitivity without detecting the magnetic field in the environment. This shield will filter the magnetic field starting from 20 Hz [17], so here we open the shield and inject a magnetic field of 4.3 nT (calibrated in 10 Hz) to the NV-cavity system to show the sensor's ability to respond to the magnetic field with different frequencies. The measured field is shown in Supplementary Figure 9(c), and it shows that the response for different frequencies varies within 6% in the range of 10 Hz to 10 kHz. This sensor response frequency test validates our claim for the sub-pT sensitivity and test field in the main text.

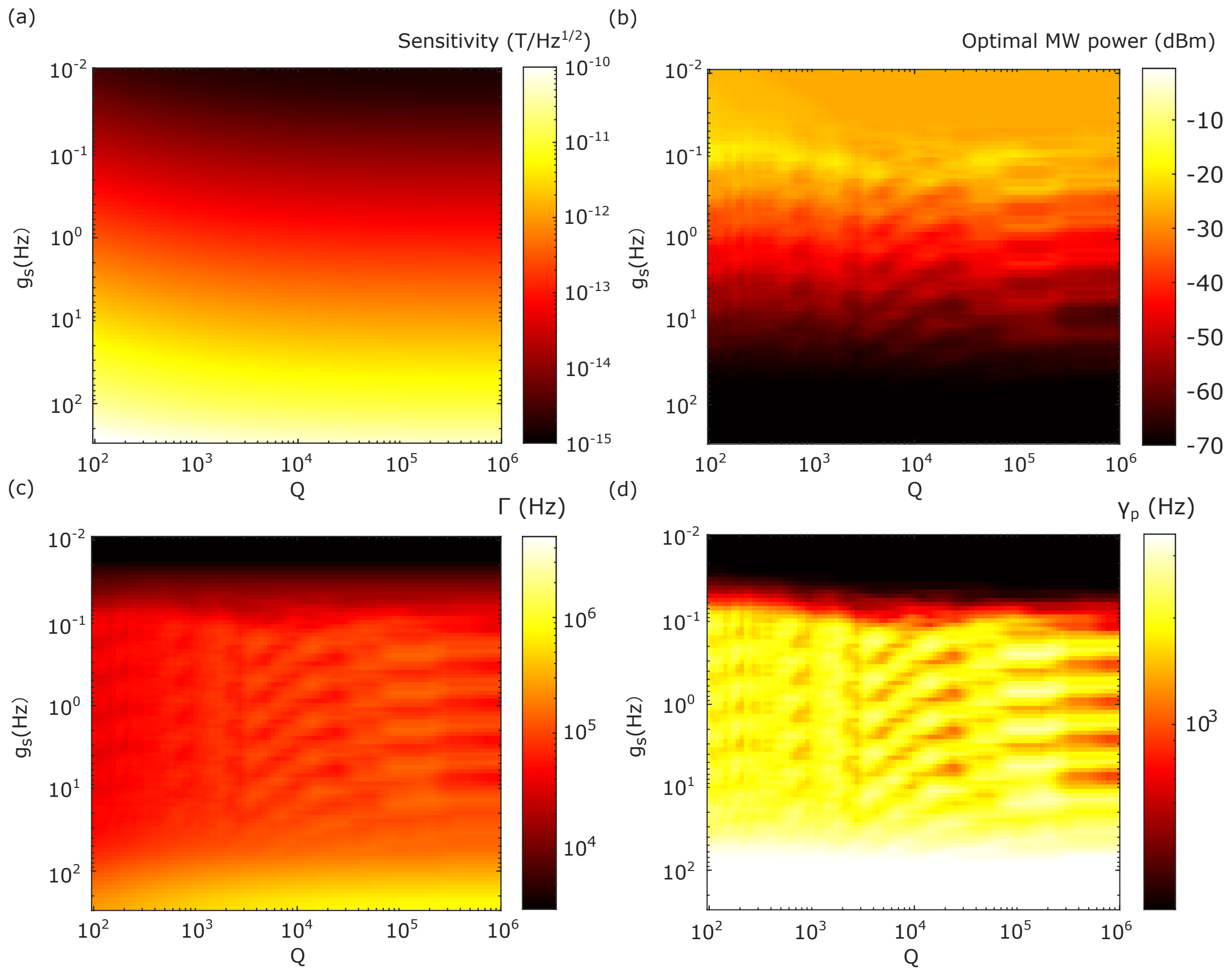

Supplementary Figure 11. Sensitivity prediction by nonlinear model. (a). Optimal sensitivity for different quality factors (Q) and single coupling strength (g_s). Optimal (b). microwave (MW) power, (c). inhomogeneous linewidth, (d). optical polarization rate for different cavity designs and coupling strength.

C. Test field

To validate our noise-spectrum-inferred sensitivity analysis, we measure a noise spectrum in the presence of a test magnetic field of known amplitude. The data from this measurement are plotted in Supplementary Figure 10(a,b). We generate the 10 kHz sinusoidal test field with $B_{\rm pp} = 5$ nT using a *in situ* coil and record the sensor response (See Methods). The difference between the measured and calculated field may be attributed to low-frequency environmental magnetic noise fields. The frequency-domain sensitivity plot with 10 kHz signal is plotted in Supplementary Figure 10(b).

V. SENSITIVITY PREDICTION

In this supplementary section we provide more information related to main text Fig. 5. In Fig. 5 we plot the optimal sensitivity for different cavity designs and diamond choice, containing quality factor Q, single coupling strength g_s, inhomogeneous linewidth Γ, NV density ρ, optical polarization rate γ_p. We bound those parameters as follows: $Q \in [10^2\ 10^6]$, $\rho \in [10^{-2}\ 10^2]$ ppm, $\Gamma/\rho = 2\pi \times 82.5$ kHz/ppm, $\gamma_0/\rho = 2\pi \times 7.5$ kHz/ppm, $\gamma_p \in 2\pi \times [0.29\ 3.43]$ kHz (maximum laser power 20 W). All the calculations and discussions below obey the optical polarization limit mentioned in the main text and Methods.

As discussed in the main text, we achieve the highest sensitivity with a high-Q cavity design combined with a low single-spin coupling strength and an optimal filling factor, as depicted in Supplementary Figure 11(a) (further discussions can be found in the main text). In Supplementary Figure 11(b), we illustrate the optimal microwave (MW)

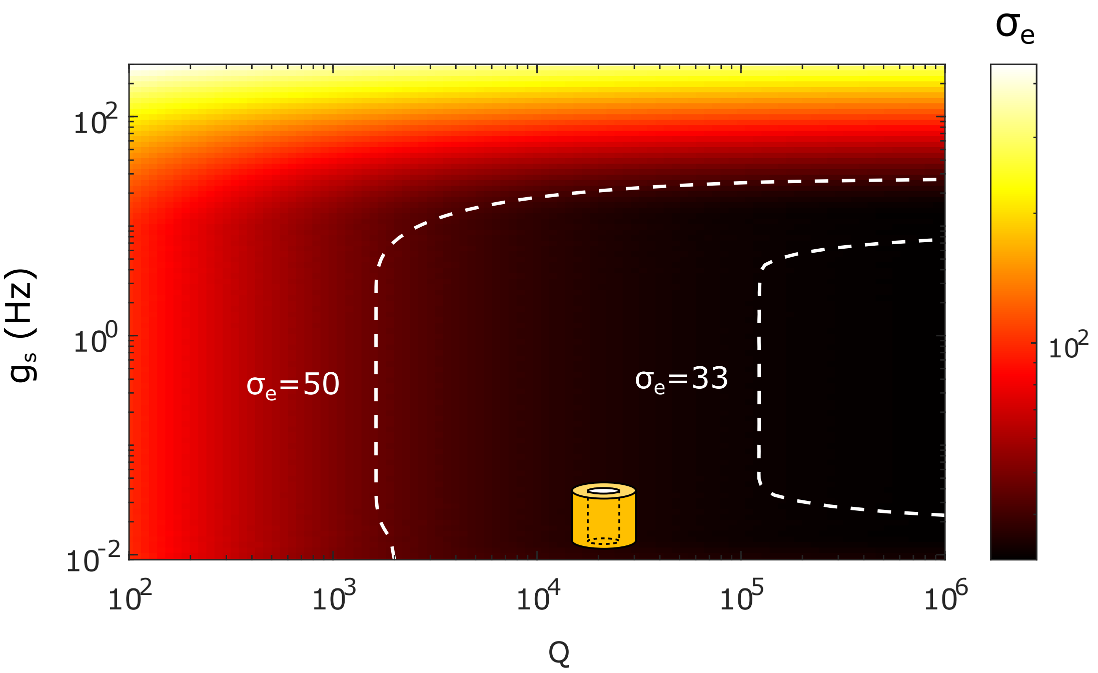

Supplementary Figure 12. Inverse readout fidelity with different quality factors and single-spin coupling strengths g_s. The inverse readout fidelity is around $\sigma_e = 33$ in high-Q and low-g_s regime.

power across different cavity-diamond parameters. We show the peak microwave power is approximately -10 dBm, and high-g_s cavity designs are associated with a lower optimal microwave power. This correlation exists because a higher g_s cavity design leads to a more pronounced quenching effect, which results in a reduced optimal MW power. In Supplementary Figure 11(c), we show the optimal inhomogeneous linewidth for varying cavity-diamond parameters. Interestingly, the optimal inhomogeneous linewidth is independent on Q at fixed frequency and critical coupling. This can be understood by our nonlinear model:

$$S = 2f(\beta_{\rm in})\frac{\chi}{\Gamma_1}\frac{C_\alpha}{(1+C_\alpha)^2}. \tag{69}$$

Based on Fig. 2, the optimal S occurs at $\chi \gg 1$. We then have $\chi \sim \sqrt{8g_s^2|\beta|^2/\gamma\gamma_{\rm p}\kappa_{\rm c1}} \propto 1/\sqrt{\kappa_{\rm c1}}$. Then the effective cooperativity $C_\alpha \propto g^2/\kappa\chi(\Gamma+\chi\gamma)$ is independent of κ at critical coupling. The other part, $\chi/\Gamma_1 = \chi/(\Gamma+\gamma\chi) \sim 1/\gamma$, is also independent of χ and κ for $\chi \gg \Gamma/\gamma$. However, S does decrease with the single coupling strength. This phenomenon can be attributed to (1) a larger inhomogeneous linewidth resulting in a weaker nonlinear quenching effect and (2) a greater inhomogeneous linewidth indicating a higher NV density, denoted as ρ, owing to the constraint $\Gamma/\rho = 82.5$ kHz/ppm. This, in turn, implies a larger total effective coupling strength. Finally, we plot the optimal optical polarization rate γ_0 in Supplementary Figure 11(d). A larger single spin coupling strength is associated with larger optical polarization rate to balance the saturation factor.

For Fig. 5, as shown in the main text, the polarizing laser light is attenuated with natural length scale $h = m_{\rm C}/\rho\sigma f \sim 0.5$ mm, where $m_{\rm C} = 2\times10^{-26}$ kg is the atomic mass of carbon, $\rho = 3.5\times10^3$ kg/m^3 is the density of diamond, and $\sigma = 7.8\times10^{-17}$ cm^2 is the absorption cross-section for excitations out of the ground state manifold [20, 21], f is the NV substitution fraction. From the nonlinear theory in the main text shown in Fig. 2(g), the sensitivity will decrease by 5% with an optical polarization decay rate of $\alpha = 3.2$. The largest thickness of the diamond h_0 can be determined by $h_0 = 2h\ln(2\alpha) \sim 2$ mm. The inner diameter of the dielectric resonator $d_0 = 6$ mm. The largest diamond volume we can put is $V_{\rm max} = \pi d_0^2 h_0/4$. We normalize the volumes with mode volume $V = \hbar\omega\gamma^2\mu_0/2g_s^2\sin^2\theta$.

The inverse readout fidelity σ_e is a useful metric for comparing different cavity designs and readout schemes. Supplementary Figure 12(b) shows the inverse readout fidelity for the optimized designs corresponding to the sensitivities shown in Fig. 5b and under the same constraints. For the current design assuming a perfect filling factor with optimal NV density, an inverse fidelity of $\sigma_e \sim 33$ may be achieved. Large device sizes are preferred in the ideal case, with the increased NV number outweighing slightly reduced fidelities. Interestingly the inverse readout fidelity is almost the same when $g_s < 2\pi\times10$ Hz. This is because although the sensitivity got improved for the low g_s cavity design, the spin projection limit is also improved, leaving the same scale between quantum limit and the cavity design. However, one can further improve the inverse readout fidelity by cooling the microwave circuit and improving the laser power.

As a comparison with previous works, the best demonstrated inverse readout fidelity for a continuous ODMR measurement is around $\sigma \sim 5000$ [11, 22, 23]. Here we experimentally demonstrate an inverse readout fidelity of 360 and a potential improvement to 33 for achievable diamond choices.

---

[1] M. Blaha, A. Johnson, A. Rauschenbeutel, and J. Volz, Beyond the tavis-cummings model: Revisiting cavity qed with ensembles of quantum emitters, Phys. Rev. A **105**, 013719 (2022).

[2] C. J. Foot, *Atomic physics*, Oxford master series in physics (Oxford University Press, Oxford ; New York, 2005).

[3] G.-Q. Zhang, Z. Chen, D. Xu, N. Shammah, M. Liao, T.-F. Li, L. Tong, S.-Y. Zhu, F. Nori, and J. Q. You, Exceptional point and cross-relaxation effect in a hybrid quantum system, PRX Quantum **2**, 020307 (2021).

[4] M. O. Scully and M. S. Zubairy, Quantum optics (1999).

[5] A. Angerer, S. Putz, D. O. Krimer, T. Astner, M. Zens, R. Glattauer, K. Streltsov, W. J. Munro, K. Nemoto, S. Rotter, J. Schmiedmayer, and J. Majer, Ultralong relaxation times in bistable hybrid quantum systems, Science Advances **3**, e1701626 (2017).

[6] Y. Zhang, Q. Wu, H. Wu, X. Yang, S.-L. Su, C. Shan, and K. Mølmer, Microwave mode cooling and cavity quantum electrodynamics effects at room temperature with optically cooled nitrogen-vacancy center spins, npj Quantum Information **8**, 125 (2022).

[7] K. Jensen, V. M. Acosta, A. Jarmola, and D. Budker, Light narrowing of magnetic resonances in ensembles of nitrogen-vacancy centers in diamond, Phys. Rev. B **87**, 014115 (2013).

[8] A. Jarmola, V. M. Acosta, K. Jensen, S. Chemerisov, and D. Budker, Temperature- and magnetic-field-dependent longitudinal spin relaxation in nitrogen-vacancy ensembles in diamond, Phys. Rev. Lett. **108**, 197601 (2012).

[9] M. L. Goldman, M. W. Doherty, A. Sipahigil, N. Y. Yao, S. D. Bennett, N. B. Manson, A. Kubanek, and M. D. Lukin, State-selective intersystem crossing in nitrogen-vacancy centers, Phys. Rev. B **91**, 165201 (2015).

[10] A. Gupta, L. Hacquebard, and L. Childress, Efficient signal processing for time-resolved fluorescence detection of nitrogen-vacancy spins in diamond, J. Opt. Soc. Am. B **33**, B28 (2016).

[11] J. F. Barry, J. M. Schloss, E. Bauch, M. J. Turner, C. A. Hart, L. M. Pham, and R. L. Walsworth, Sensitivity optimization for nv-diamond magnetometry, Rev. Mod. Phys. **92**, 015004 (2020).

[12] R. Chapman and T. Plakhotnik, Quantitative luminescence microscopy on nitrogen-vacancy centres in diamond: Saturation effects under pulsed excitation, Chemical Physics Letters **507**, 190 (2011).

[13] A. Dréau, M. Lesik, L. Rondin, P. Spinicelli, O. Arcizet, J.-F. Roch, and V. Jacques, Avoiding power broadening in optically detected magnetic resonance of single nv defects for enhanced dc magnetic field sensitivity, Phys. Rev. B **84**, 195204 (2011).

[14] M. Wang, M. Caouette-Mansour, A. Solyom, and L. Childress, Comparing continuous and pulsed nitrogen-vacancy dc magnetometry in the optical-power-limited regime, J. Opt. Soc. Am. B **41**, 62 (2024).

[15] E. Bauch, S. Singh, J. Lee, C. A. Hart, J. M. Schloss, M. J. Turner, J. F. Barry, L. M. Pham, N. Bar-Gill, S. F. Yelin, and R. L. Walsworth, Decoherence of ensembles of nitrogen-vacancy centers in diamond, Phys. Rev. B **102**, 134210 (2020).

[16] R. . Schwarz, Rohde & schwarz sma100b signal generator documentation (2023).

[17] R. Wilcox, E. Eisenach, J. Barry, M. Steinecker, M. O'Keeffe, D. Englund, and D. Braje, Thermally polarized solid-state spin sensor, Phys. Rev. Appl. **17**, 044004 (2022).

[18] E. Ivanov, M. Tobar, and R. Woode, Microwave interferometry: application to precision measurements and noise reduction techniques, IEEE Transactions on Ultrasonics, Ferroelectrics, and Frequency Control **45**, 1526 (1998).

[19] D. P. Fahey, K. Jacobs, M. J. Turner, H. Choi, J. E. Hoffman, D. Englund, and M. E. Trusheim, Steady-state microwave mode cooling with a diamond n-v ensemble, Phys. Rev. Appl. **20**, 014033 (2023).

[20] S. R. Nair, L. J. Rogers, X. Vidal, R. P. Roberts, H. Abe, T. Ohshima, T. Yatsui, A. D. Greentree, J. Jeske, and T. Volz, Amplification by stimulated emission of nitrogen-vacancy centres in a diamond-loaded fibre cavity, Nanophotonics **9**, 4505 (2020).

[21] T.-L. Wee, Y.-K. Tzeng, C.-C. Han, H.-C. Chang, W. Fann, J.-H. Hsu, K.-M. Chen, and Y.-C. Yu, Two-photon excited fluorescence of nitrogen-vacancy centers in proton-irradiated type ib diamond, The Journal of Physical Chemistry A **111**, 9379 (2007), pMID: 17705460.

[22] J. M. Schloss, J. F. Barry, M. J. Turner, and R. L. Walsworth, Simultaneous broadband vector magnetometry using solid-state spins, Phys. Rev. Appl. **10**, 034044 (2018).

[23] J. F. Barry, M. J. Turner, J. M. Schloss, D. R. Glenn, Y. Song, M. D. Lukin, H. Park, and R. L. Walsworth, Optical magnetic detection of single-neuron action potentials using quantum defects in diamond, Proceedings of the National Academy of Sciences **113**, 14133 (2016).
